# Pod Morphology, Primary and Secondary Metabolite Profiles in Non-grafted and Grafted Carob Germplasm Are Configured by Agro-Environmental Zone, Genotype, and Growing Season

**DOI:** 10.3389/fpls.2020.612376

**Published:** 2021-01-13

**Authors:** Angelos C. Kyratzis, Chrystalla Antoniou, Lambros C. Papayiannis, Giulia Graziani, Youssef Rouphael, Marios C. Kyriacou

**Affiliations:** ^1^Agricultural Research Institute, Ministry of Agriculture, Rural Development and Environment, Nicosia, Cyprus; ^2^Department of Pharmacy, University of Naples Federico II, Naples, Italy; ^3^Department of Agricultural Sciences, University of Naples Federico II, Portici, Italy

**Keywords:** functional quality, genetic diversity, polyphenols, SSRs, sugars, tannins

## Abstract

Carob is a predominantly rainfed tree crop of high nutritive value and a long history of adaptation to the edaphoclimatic stress conditions of the Mediterranean. However, declining attention to the carob tree in recent decades has aggravated genetic erosion. The extant *in situ* germplasm varies both in terms of pod morphology and composition, reflecting the genetic and physiological divide chiefly among grafted and non-grafted material, and possibly the impact of variable agro-environments. Accordingly, the present study aimed to establish a systematic categorization of the genetic and phenotypic diversity encountered across carob germplasm identified *in situ* throughout Cyprus, a historical center of production and genetic diversity for the species. Linking pod morphology, primary and secondary metabolite profiles with genotyped source material originating in different agro-environments and crop seasons would provide a framework for interpreting (a) the interaction of these factors in configuring carob pod physicochemical constitution, and (b) the relative stability of phenotypic traits against environmental and seasonal variation. Microsatellite analysis discriminated 36 genotypes out of the 124 trees located in nine traditional agro-environmental zones and revealed low genetic diversity within the grafted germplasm. Two landraces were identified: “Tillyria,” which is widespread and predominant, and “Kountourka,” which is mainly localized to the northeastern peninsula of Karpasia. Morphological traits, such as seeds-to-pod weight ratio, pod width and thickness were principally under genetic control. Contrarily, compositional traits, particularly total phenolic content—including condensed tannins, *in vitro* antioxidant capacity and to a lesser extent gallic acid, organic acids and minerals were under agro-environmental control. Agro-environmental zone also modulated principally fructose and glucose; sucrose was modulated equally by genotype and agro-environment, while total sugars were under genetic control. Statistically significant differences between seasons were detected for all traits except for the seeds-to-pod weight ratio, pod length and width. Hierarchical cluster analysis corroborates that Cyprus may be divided into two major agro-environmental zones modulating the compositional properties of the carob pulp. The present study provides a comprehensive insight into the extant carob genetic resources of Cyprus and advances our understanding of how genetic, agro-environmental and seasonal factors interact in shaping carob pod morphology and composition.

## Introduction

Carob (*Ceratonia siliqua* L.) is an evergreen, diploid species (2n = 48) that belongs to the *Fabaceae* family. The carob tree is an important component of the Mediterranean vegetation and it is predominately cultivated in dry and marginal areas, due to its low requirements in agronomic inputs compared to other fruit species. Its ability to thrive in the prevailing calcareous soils has amplified the socio-economic value of the carob in several Mediterranean mild climate dry lands (Batlle and Tous, 1997; Tous et al., 2013).

The carob tree occurred in the flora of the East Mediterranean basin long before the emergence of agriculture and it was naturally distributed westwards to the rest of Mediterranean countries (Zohary, 2002; Viruel et al., 2016). The domestication and cultivation of carob appeared relatively late in Hellenistic and Roman times as vegetative propagation was not effective for carob propagation and only after the discovery of scion grafting have selected phenotypes been cultivated. It is nonetheless worth noting that cultivation essentially comprised naturally and randomly distributed wild rootstocks grafted with select scion phenotypes rather than systematic orchards, which only came about in recent decades (Tous et al., 2013). Grafted carob trees differ from their wild ancestors mainly in phenotypic traits such as fleshiness, size and sweetness of the pod, local adaption and productivity (Batlle and Tous, 1997; Zohary, 2002; Tous et al., 2013).

For centuries, the carob fruit has been used as a regional culinary ingredient and as a livestock feed due to its high sugar content (Batlle and Tous, 1997). Nowadays, carob cultivation is expanding in response to growing demand for its compositional, functional, nutritional, and industrial value which makes it an economically important crop (Goulas et al., 2016; Stavrou et al., 2018). The mature carob pod is comprised of two parts: the pulp and the seeds in roughly 90/10 ratio w/w (Goulas et al., 2016). Carob seeds are exploited industrially for the production of carob bean gum (Locust Bean Gum—LBG), a widely used natural food thickening agent (Bouzouita et al., 2007). Recently, researchers have focused on carob pulp which is a low-cost by-product of the milling process. Carob pod is not only a rich source of sugars but also of bioactive molecules including dietary fibers, polyphenols and cyclitols and it has a low-fat content (Avallone et al., 1997). These bioactive compounds exhibit a wide range of biological properties with significant health-promoting effects, including the prevention of colon cancer and hepatocellular carcinoma, reduction of diarrheal symptoms, lowering of LDL cholesterol as well as antidiabetic effects (Zunft et al., 2003; Goulas et al., 2016; Theophilou et al., 2017). These findings have contributed to the valorization of carob pulp products such as carob powder, fiber, juice and molasses used by the food industry for developing a wide range of health-promoting or niche food products, including gluten-free ones (Yousif and Alghzawi, 2000; Nasar-Abbas et al., 2016).

The carob constitutes a genetic resource of long-standing adaptation to the edapho-climatic conditions of Cyprus, which has been for centuries one of the leading countries in carob production (Ticho, 1959; Orphanos, 1980). Carob pods produced from specific geographical areas of Cyprus are considered of premium quality and they are customarily exported intact to Egypt for human consumption (Gennadius, 1902; Personal communication with local stakeholders 2018, 2019). Based on morphological traits (Orphanos and Papaconstantinou, 1969) and other records (Gennadius, 1902; Ticho, 1959; Davies, 1970), the Cypriot carob germplasm can be categorized into four groups. The first group comprises wild trees producing short and thin pods of variable morphology and substandard quality, which farmers historically did not harvest. The second group contains non-grafted trees producing pods of reasonably good quality, identified by farmers as “Apostolika,” a term alluding to their possible use as foodstuff by Christ’s wandering apostles. Scattered trees of this group can be found within carob groves and farmers invariably collect their pods. The third group, which predominates on the island, encompasses exclusively grafted trees described in the literature as “Tillyria,” reference to the prolific geographical area of the island’s central northern coast (Gennadius, 1902). The name “Tillyria,” however, is not ubiquitous among farmers, which instead tend to identify these trees under several local names. “Tillyria” produce pods of slightly variable morphology, and it remains unanswered if this variation is due to genetic variability or due to the variability of edapho-climatic conditions. The fourth group contains grafted trees producing shorter pods than “Tillyria,” locally common to the Karpasia peninsula and referred to as “Kountourka.” In addition, two scion phenotypes, morphologically proximate to “Tillyria,” have been reported. These phenotypes are called “Mavroteratsia” and “Koumpota.”

The preferential cultivation of irrigated cash crops (e.g., citrus) after the 1960s in Cyprus led to the depreciation of the carob crop and resulted in substantial reduction of the carob cultivated area (Davies, 1970). Moreover, wild fires, illegal logging and heavy rat infestation have further threatened the species’ diversity (Orphanos, 1980). The preference of farmers for a specific select phenotype, in the case of Cyprus for “Tillyria,” could pose an additional threat for the species’ genetic diversity (Barracosa et al., 2007). However, the reviving interest in the carob warrants the investigation of its genetic diversity to establish conservation and breeding programs (Di Guardo et al., 2019). Further to the classical studies on genetic diversity based on morphological traits (Barracosa et al., 2007), the use of molecular markers caries the advantage that they are more polymorphic and unaffected by the environment. Microsatellites (SSRs) have been successfully used to assess carob genetic diversity (La Malfa et al., 2014; Viruel et al., 2018; Di Guardo et al., 2019). Additionally, a prerequisite for revitalizing the carob industry is the assessment of the variation for morphological and compositional traits (Barracosa et al., 2007; Custodio et al., 2011; Benchikh et al., 2014). However, for rain fed crops, such as carob, grown under marginal environments, the agro-environmental effect owing to spatial variation in edapho-climatic conditions would be expected to predominate over the genotypic effect (Blum, 2010). Furthermore, annual variation in climatic conditions may have a significant effect on productivity (Orphanos, 1980), hence putatively also on compositional traits. However, very few studies have investigated the environmental effect on carob compositional traits (Avallone et al., 1997; El Bouzdoudi et al., 2016; Farag et al., 2019; Othmen et al., 2019) while information concerning the seasonal effect on compositional traits remains scarce (Nahla, 2014; Correia et al., 2018).

The current study combined genetic and phenotypic data collected from trees *in situ/on farm* to dissect the genotypic and the agro-environmental effects on morphological and compositional traits. Extensive sampling was performed from all the traditional environmental zones of carob cultivation, with emphasis on grafted material. Sampling was performed for two consecutive years to evaluate the seasonal effect on phenotypic traits. The genetic and phenotypic variation of Cypriot carob genetic resources is hereby presented. To the best of our knowledge, this is the first study aiming to investigate the genotypic, agro-environmental and seasonal effects on carob morphological and compositional traits.

## Materials and Methods

### Sampling Strategy

**FIGURE 1:**
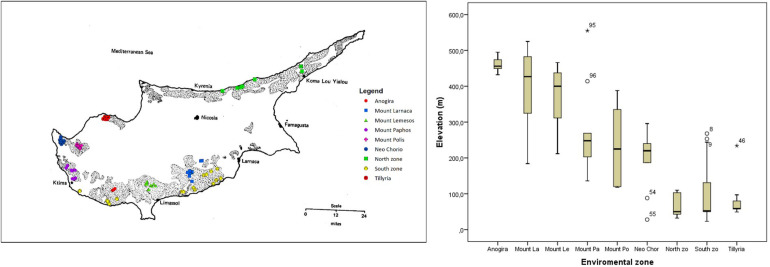
Map of Cyprus showing the extent of sampling in the current study. Dashed pattern presents the traditional areas of carob cultivation (after Davies, 1970). Sampled trees in different agro-environmental zones are depicted in different color. Box plot shows the variation in altitude of sampled trees within the different agro-environmental zones.

Extensive survey of carob genetic resources was carried out during two seasons, 2018 and 2019. Trees were sampled from nine agro-environmental zones based on geographical and geological parameters (Figure 1). “Anogira,” “Mountainous Larnaca,” and “Mountainous Lemesos” were the inland zones of relatively high altitude. “Mountainous Paphos,” “Mountainous Polis,” and “Neo Chorio” were the zones of intermediate altitude, with the latter positioned closer to the sea than the two former zones. “Tillyria,” “North zone,” and “South zone” were the zones of low altitude where the majority of trees are grown relatively close to the sea. Trees in the “Tillyria” zone are grown on igneous formations with pillow lavas and diabase dikes while trees in the other zones are grown on calcareous formations and to lesser extent on red soils overlying a white soft highly calcareous layer. Average annual precipitation ranges between 400 and 600 mm across zones.

Vernacular knowledge on phenotypic diversity, cultivation practices, trade and processing were collected through interviews with farmers and stakeholders of the local industry. Farmers participated in joint field trips to locate trees of distinct phenotypic traits. Non-grafted trees were also sampled to assess the relatedness between grafted and non-grafted gene pools. Additionally, trees from the nursery plantations of the Department of Agriculture, Ministry of Agriculture, Rural Development and Environment (DOA) which provide budding wood for propagation purposes were sampled. Three accessions, representing the cultivated material in Sicily, were also included in the study. In total, 124 trees were sampled out of which 107 were grafted and 17 non-grafted. The trees were georeferenced and passport data were compiled (Supplementary File 1). Leaves were collected for DNA extraction. Around 30–40 carob pods were randomly harvested from each tree to assess morphological and compositional traits. Harvesting was performed during the major harvesting period (mid-August to mid-September) when pods were fully mature. The exact harvest date for each tree was recorded (Supplementary File 1). Sampling was repeated for two consecutive years with the exception of non-bearing trees due to severe pruning (trees from the DOA plantations), extensive damage from rats, or trees that were identified during the 2nd season of the survey.

### Genetic Analysis

DNA was extracted using the DNeasy Plant Mini Kit (Qiagen, Venlo, Netherlands). DNA integrity was verified in agarose electrophoresis and DNA quality and quantity was determined by NanoDrop 1000 (Thermo Fisher Scientific, Wilmington, United States). Eighteen microsatellites were selected based on the available information from previous studies (La Malfa et al., 2014; Viruel et al., 2018). Amplification reactions were set up in a 12 μL volume of a mixture containing 50 ng of genomic DNA, 1x Type-it Multiplex PCR master mix (Type-it Microsatellite PCR kit, Qiagen, Venlo, Netherlands) and 0.2 μM of each primer (the forward primers were 5′-end labeled with FAM—5-and HEX carboxy-fluorescents). PCR amplification was performed in a PTC-200 thermocycler (Bio-Rad, Hercules, United States) under the following temperature profile: 5 min at 95°C, followed by 30 cycles, each one including 30 s at 95°C, 30 s at annealing temperature depending of the primer pair (Supplementary File 2), 30 s at 72°C and a final extension step for 30 min at 60°C. A negative control was included in each set of PCR amplification. A subset of accessions (10%) was amplified twice to check the reproducibility of the primers. Amplified PCR products were run on an ABI3130 genetic analyzer (Applied Biosystems, Foster City, CA, United States). Size standard GeneScan^TM^ 500LIZ (Applied Biosystems) was added to each sample to delineate allele sizes. Data were analyzed using GeneMapper Software version 4.1 (Applied Biosystems, Foster City, CA, United States).

### Morphological Characterization and Processing of Plant Material

Carob pods were washed under tap water to remove debris, rinsed with deionized water and patted dry. The thickness of the pod (determined lengthwise in three sections: edge, groove, valley) and the length and width of the pod were recorded using a caliber. Then the pods were coarsely ground in a Vita Prep 3 blender (Vita-Mix Corp., Cleveland, United States) operated at low speed and deseeded. Pod and seed weight were measured using a Precisa XT120A analytical balance (Precisa Gravimetrics, Dietikon, Switzerland). The carob kibbles were lyophilized in a Christ, Alpha 1–4 lyophilizer (Osterode, Germany) to stable weight, ground to powder of 1–2 mm granulometry using a CT293 Cyclotech mill (Foss Analytical A/S, Hillerød, Denmark) and stored at −60°C.

The color lightness (L^∗^) of the carob pod and seed were assessed using a Minolta CR-400 Chroma Meter (Minolta, Osaka, Japan) and that of the powder using a Minolta CR-410 both with a diffusion illumination 0° viewing angle geometry and the color space XYZ, Yxy, L^∗^a^∗^b^∗^, Hunter, L^∗^C^∗^h, Munsel as the default.

### Minerals, Organic Acids, and Protein Content

Analysis of the cations (K^+^, Ca^2+^, Mg^2+^, Na^+^, and NH_4_^+^), anions (NO^–3^, PO_3_^–4^, SO_4_^–2^, and Cl^–^) and organic acids (malic, citric and oxalic) on lyophilized carob powder was performed as previously described in detail by Rouphael et al. (2017). The monovalent and bivalent cations were separated by ion chromatography (ICS-3000, Dionex, Sunnyvale, CA, United States) and quantified through an electrical conductivity detector. Cations separation was performed using an IonPac CG12A (4 × 50 mm, Dionex, Corporation) guard column and IonPac CS12A (4 × 250 mm, Dionex, Corporation) analytical column, whereas for anions and organic acids an IonPac AG11-HC guard (4 × 50 mm) column and IonPac AS11-HC analytical column (4 × 250 mm) were used. Minerals and organic acids content results were expressed in g kg^–1^ dw. The total protein content was assessed by the Kjeldahl method, with nitrogen-to-protein conversion factor of 6.25 (Bremner, 1965). Analysis of cations, organic acids and protein content was performed only on samples harvested in 2018.

### Total Phenols and Condensed Tannins

The total phenols content (TPC) of the carob powder was determined according to the method of Singleton et al. (1999), slightly modified as previously described by Kyriacou et al. (2016). Approximately 0.1 g of powder was extracted in solvent to a final volume of 25 mL. Extraction was performed in the dark at 4°C for 24 h. Two different extraction solvents were used: (a) methanol:H_2_O:HCl (50:40:10); and (b) methanol:H_2_O:acetate (80:19.5:0.5). In addition to the extraction of readily extractable phenolics, the former solution facilitated the extraction of acid-hydrolyzed condensed tannins. Absorbance was measured on a Jasco V-550 UV–vis spectrophotometer (Jasco Corp., Tokyo, Japan) and quantification was performed using linear calibration with external gallic acid standards over the range of 50-500 mg L^–1^, yielding a regression coefficient *R*^2^ > 0.99. TPC was expressed as gallic acid equivalents in g kg^–1^ pulp dw.

Condensed tannins (proanthocyanidins) were determined using a modification of the vanillin method described by Sun et al. (1998) and Sepperer et al. (2019). Extraction was performed as described above for phenolics using solvent (a) on 0.5 g of lyophilized powder to a final volume of 10 mL. One milliliter of extract was combined with 2.5 mL of 2% vanillin methanolic solution in 15 mL Falcon tubes placed on ice; then 2.5 mL of MeOH:HCl (90:10) were added, swirled and kept on ice for 5 min, followed by 15 min incubation at 30°C with gentle agitation (80 rpm). Absorbance was measured at 500 nm against calibration with seven catechin standards (0.025–0.5% w/v in methanol) replacing the sample. Methanol was used in the place of sample or standard as blank. The results were expressed in catechin equivalents as mg g^–1^ pulp dw.

### Analysis of Polyphenols by UHPLC-Q-Orbitrap HRMS

Polyphenols were determined on methanolic extracts obtained using the extraction solvent (b) described above. An Ultimate 3000 UHPLC system (Thermo Fisher Scientific, Waltham, MA, United States) was employed equipped with a Kinetex 1.7 μm biphenyl (100 × 2.1 mm) column (Phenomenex, Torrance, CA, United States) maintained at 25°C. The injection volume was 2 μL and flow rate was 0.2 mL min^–1^. The mobile phase consisted of water (A) and methanol (B), both containing 0.1% formic acid. Gradient elution program was applied as follows: 0 min—5% B, 1.3 min—30% B, 9.3 min—100% B, 11.3 min—100% B, 13.3 min—5% B, 20 min—5% B. The UHPLC system was coupled to a Q Exactive Orbitrap liquid chromatography tandem-mass spectrometry (LC–MS/MS). A heated electrospray ionization source (HESI II, Thermo Fischer Scientific) operating in negative ion mode (ESI^–^) was used. Ion source parameters were: sheath gas-flow rate 45 units, auxiliary gas-flow rate 10 units, spray voltage −3.5 kV, capillary temperature 275°C, S-lens (RF) level 50, auxiliary gas heater temperature 350°C.

Analysis was arranged setting two scan events (Full ion MS and All ion fragmentation, AIF) for all compounds of interest. Full MS data were acquired setting the following parameters: microscans, 1; AGC target, 1e6; maximum injection time, 200 ms; mass resolution, 35,000 FWHM at m/z 200 and m/z range, 80–1,200. AIF scan conditions were: microscans, 1; AGC target, 1e5; maximum injection time, 200 ms; mass resolution, 17,500 FWHM at m/z 200; HCD energy, 10, 20, and 45 and m/z range, 80–1,200. Calibration of the Q Exactive Orbitrap LC–MS/MS was checked daily, in both negative and positive modes, using the commercial calibration solutions provided by the manufacturer. Mass tolerance was kept at 5 ppm in both fullscan MS and AIF modes. Xcalibur software v. 3.1.66.10 (Xcalibur, Thermo Fisher Scientific) was used to perform data analysis and processing. Single phenolic compounds were quantified using calibration curves built with appropriate standards. As some standards were not available, quantitation for some compounds was carried out employing calibration curves of available standard belonging to the same chemical group and with similar response to the mass spectrometer. Analysis of polyphenols by UHPLC-Q-Orbitrap HRMS was performed only on samples harvested in 2018.

### *In vitro* Antioxidant Activity

The *in vitro* Ascorbate Equivalent Antioxidant Activity (AEAC) of carob powder was assayed on pulp methanolic extracts according to the 1,1-diphenyl-2-picrylhydrazyl free-radical (DPPH) scavenging capacity assay of Brand-Williams et al. (1995) modified according to Kyriacou et al. (2020). Quantification was performed against ascorbate external standards (100–1,000 μM) based on the decrease in absorbance at 517 nm and expressed in ascorbate equivalents as g kg^–1^ pulp dw. The *in vitro* antioxidant activity of carob powder methanolic extracts was assayed also according to the ferric reducing antioxidant power (FRAP) assay of Benzie and Strain (1996). Quantification was performed at 593 nm against external standard curves of ascorbic acid at 100–1,000 μM. Results were expressed in ascorbate equivalents as g kg^–1^ pulp dw.

### Water Soluble Carbohydrates

The analysis of water-soluble carbohydrates was performed on aqueous extracts clarified using the Carrez Clarification Kit (Sigma-Aldrich, St. Louis, MO, United States). Separation and quantification of glucose, fructose and sucrose was performed by liquid chromatography on an Agilent HPLC system (Agilent Technologies, Santa Clara, CA, United States) equipped with a 1,200 Series quaternary pump and a 1,260 Series Refractive Index detector operated by Chem-Station software as previously described by Kyriacou et al. (2016). Injection volume was 20 μL and separation was performed on a Waters 4.6 × 250 mm carbohydrate column (Waters, Milford, MA, United States) at 35°C using an acetonitrile:water (82:18) mobile phase at an isocratic flow rate of 1.5 mL min^–1^. Quantification was performed against calibration curves of fructose, glucose and sucrose external standards (0.2–2.0 g 100^–1^ mL) with a coefficient of determination *R*^2^ > 0.999 and expressed as g 100 g^–1^ pulp dw.

### Statistical Analysis

Genetic analysis was conducted on allele fragments. Three out of the eighteen primers (Cesi 21 cttt7, C21, and C23) amplified more than two alleles per tree sample, therefore, in the analysis, it was assumed that these primers were multilocus. Number of alleles (Na), number of alleles with frequency less than five (N ≤ 0.05), number of private alleles, effective number of alleles (Ne), observed (Ho) and expected heterozygosity (He), fixation index (Fis) and deviation for Hardy Weinberg equilibrium (HW sign) were calculated on the whole set of trees and separately for grafted and non-grafted trees using GenAlEx 6.4 (Peakall and Smouse, 2006). Polymorphic information content (PIC) and probability for null alleles F (null) were estimated with Cervus ver. 3.0.7 (Kalinowski et al., 2007). Analysis of Molecular Variance (AMOVA) was also performed to assess the within and between variance across grafted and non-grafted trees using GenAlEx. The significance of the resulting variance components and the inter-population genetic distances were tested using 999 random permutations. Simple matching dissimilarity index was used to calculate genetic distances between tree pairs and a weighted Neighbor-Joining dendrogram was constructed using the DARwin software, version 6 (Perrier, 2006). The robustness of the tree was tested using 10,000 bootstraps and bootstrapped values above 20% are presented. STRUCTURE software (ver. 2.3.4) was employed to investigate the genetic structure using the admixture model with 100,000 burn in followed by 100,000 iterations with 30 replicate runs (Pritchard et al., 2000). Ten clusters were tested (*k* = 1–10) and the validation of the most likely number of clusters K was performed with the Structure Harvester following the ΔK method proposed by Evanno et al. (2005). Trees were assigned to a cluster when the membership coefficient was ≥ 0.8 (Di Guardo et al., 2019).

Mean values, standard error of means, coefficients of variation, maximum and minimum values were calculated and frequency plots were constructed for all morphological and compositional traits. Two tailed *t*-tests were conducted to explore trait differences between seasons and Pearson correlations were estimated to investigate their relationships. Separate Analyses of Variance (ANOVA) were conducted to investigate the genetic and agro-environmental effects on the examined traits. The first ANOVA was executed using as factor treatments the clustering of the trees according to the structure analysis, while the second ANOVA was executed using as factor treatments the agro-environmental zones where the trees are grown. The percentage of variance explained by the main effect, as percentage of the SS to the total SS, is presented. Mean values and standard error of the means were calculated for each structure cluster and agro-environmental zone. Box plots are presented in cases where the percentage of variance explained by the genotypic or agro-environmental effect was particularly high. Hierarchical cluster analysis was conducted to assess the genetic and agro-environmental effects on the overall phenotype. Squared Euclidean distances were calculated on standardized *Z*-values, with a mean of 0 and a standard deviation of 1. Clustering was performed using the “WARD” method. All analyses were carried out using SPSS (IBM, SPSS ver. 26).

## Results

### Genetic Variation and Affinity Across Trees

**FIGURE 2:**
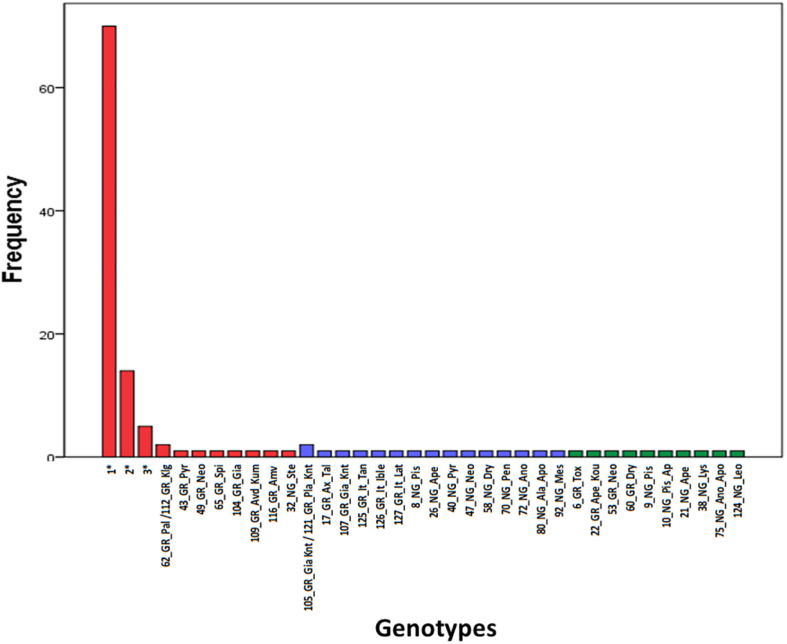
Frequency of appearance of the 36 genotypes. Genotypes grouped into clusters (G) and (N) of the structure analysis are shown in red and blue color, respectively. Admixture (A) genotypes are shown in green color. The first component of the id code refers to tree number, the second to grafted (GR) or non-grafted trees (NG), the third to tree location and the fourth to the variety identification by farmers. Information concerning multiple tress assigned to genotypes 1, 2, and 3 can be found in Supplementary File 1.

Microsatellite analysis was used to genotype carob trees from nine agro-environmental zones. The 18 primer pairs used in the present study discriminated 36 genotypes. The primers distinguished all non-grafted trees. Moreover, 20 genotypes were identified among the 107 grafted trees. The most common genotype appeared with very high frequency (Figure 2). In total, 86 alleles were detected, out of which 49 appeared with low frequency and 15 where private. Average, Ho, He, PIC and Fis, where 0.547, 0.354, 0.295, and −0.319, respectively. For most loci, there was significant deviation from Hardy Weinberg equilibrium. The probability for null alleles was low (Table 1). Genetic diversity indices for grafted and non-grafted trees are shown in Table 2. Ho was higher for grafted trees and the number of alleles detected was equal, while the number of private alleles and He were higher for non-grafted trees. Contrary to grafted trees, Fis was close to zero and most primers did not show significant deviation from Hardy Weinberg equilibrium for non-grafted trees. Primers C21 and C23 depicted higher polymorphism and discriminating ability within both groups. AMOVA showed that 32% of variance accounted for variation between grafted and non-grafted trees, while the remaining 68% was due to variance within each group. There was significant divergence between grafted and non-grafted trees (*PhiPT* = 0.321, *p* = 0.01), and Nm value was 0.528.

**TABLE 1:** Genetic diversity indices, deviation from Hardy Weinberg equilibrium (HW sign) and probability for null alleles F(null) among the trees sampled.

Locus	Na	Range	N ≤ 0.05	Private	Ne	Ho	He	PIC	Fis	HW sign	F(null)
Cesi 21 cttt7 L1	2	183–191	0	0	1.971	0.863	0.493	0.371	–0.751	***	–0.273
Cesi 21 cttt7 L2	2	280–288	0	0	1.994	0.927	0.498	0.374	–0.861	***	–0.301
Cesi 187 at15	6	139–158	4	1	2.330	0.952	0.571	0.478	–0.667	***	–0.267
Cesi 1187 at9	5	152–175	3	2	2.145	0.927	0.534	0.426	–0.737	***	–0.280
Cesi 98 gct6	3	161–171	1	0	2.149	0.895	0.535	0.429	–0.674	***	–0.264
Cesi 15 aaatag4	2	315–320	1	0	1.084	0.048	0.077	0.074	0.375	***	0.217
Cesi 976 ta5tg6	3	249–255	2	0	1.067	0.056	0.063	0.062	0.103	**	0.106
Cesi 74 ta7	2	277–279	1	0	1.093	0.073	0.085	0.081	0.144	ns	0.077
Cesi 17 tta7	2	170–173	0	0	1.990	0.831	0.497	0.374	–0.670	***	–0.251
C8	6	239–270	5	1	1.254	0.185	0.203	0.198	0.086	***	0.091
C10	3	211–227	1	0	2.092	0.879	0.522	0.412	–0.684	***	–0.264
C22	3	223–239	1	0	1.218	0.105	0.179	0.170	0.413	***	0.272
C23 L1	2	196–198	1	0	1.033	0.032	0.032	0.031	–0.016	ns	–0.002
C23 L2	12	209–262	9	4	3.166	0.893	0.684	0.632	–0.306	***	–0.167
C29	3	151–159	1	0	2.016	0.903	0.504	0.385	–0.792	***	–0.285
C31	3	240–248	2	1	1.025	0.024	0.024	0.024	–0.009	ns	–0.001
C4	5	191–209	2	0	2.425	0.976	0.588	0.501	–0.661	***	–0.267
C5	4	146–163	3	1	1.140	0.121	0.123	0.120	0.016	***	0.029
C21 L1	2	160–163	1	0	1.033	0.032	0.032	0.031	–0.016	ns	–0.002
C21 L2	12	174–231	9	4	3.166	0.893	0.684	0.632	–0.306	***	–0.167
C33	4	231–243	2	1	2.059	0.871	0.514	0.398	–0.694	***	–0.261
Mean	4.095		2.333	1.000	1.783	0.547	0.354	0.295	–0.319		–0.108
Total	86		49	15							

Na, Number of alleles; N ≤ 0.05, number of alleles with frequency less than five; Private, number of private alleles; Ne, effective number of alleles; Ho, observed heterozygosity; He, expected heterozygosity; PIC, polymorphic information content; Fis, fixation index; ns, non-significant **P < 0.01, ***P < 0.001.

**TABLE 2:** Genetic diversity indices and deviation from Hardy Weinberg equilibrium (HW sign) among the grafted and the non-grafted trees.

	Grafted								Non-grafted							
Locus	Na	N ≤ 0.05	Private	Ne	Ho	He	Fis	HW sign	Na	N ≤ 0.05	Private	Ne	Ho	He	Fis	HW sign
Cesi 21 cttt7 L1	2	0	0	1.989	0.925	0.497	–0.861	***	2	0	0	1.710	0.471	0.415	–0.133	ns
Cesi 21 cttt7 L2	2	0	0	2.000	0.981	0.500	–0.963	***	2	0	0	1.710	0.588	0.415	–0.417	ns
Cesi 187 at15	5	3	1	2.196	0.981	0.545	–0.802	***	5	1	1	3.211	0.765	0.689	–0.111	ns
Cesi 1187 at9	4	2	1	2.056	0.972	0.514	–0.892	***	4	1	1	2.569	0.647	0.611	–0.059	ns
Cesi 98 gct6	3	1	0	2.110	0.953	0.526	–0.812	***	3	0	0	2.149	0.529	0.535	0.010	ns
Cesi 15 aaatag4	2	1	0	1.048	0.028	0.046	0.386	***	2	0	0	1.335	0.176	0.251	0.297	ns
Cesi 976 ta5tg6	3	2	1	1.038	0.037	0.037	–0.015	ns	3	0	0	1.273	0.176	0.215	0.177	ns
Cesi 74 ta7	2	1	0	1.028	0.028	0.028	–0.014	ns	2	0	0	1.562	0.353	0.360	0.019	ns
Cesi 17 tta7	2	0	0	2.000	0.935	0.500	–0.869	***	2	0	0	1.637	0.176	0.389	0.547	*
C8	6	5	2	1.132	0.103	0.117	0.120	***	5	0	0	2.546	0.706	0.607	–0.162	ns
C10	3	1	0	2.031	0.944	0.508	–0.859	***	3	0	0	2.181	0.471	0.542	0.131	ns
C22	3	2	0	1.088	0.019	0.081	0.770	***	3	0	0	2.379	0.647	0.580	–0.116	ns
C23 L1	2	1	1	1.009	0.009	0.009	–0.005	ns	2	0	0	1.192	0.176	0.161	–0.097	ns
C23 L2	10	7	3	2.936	0.944	0.659	–0.431	***	10	4	6	5.294	0.533	0.811	0.342	***
C29	3	1	1	2.015	0.953	0.504	–0.892	***	3	1	1	1.835	0.588	0.455	–0.293	ns
C31	1	0	0	1.000	0.000	0.000			3	1	1	1.197	0.176	0.164	–0.074	ns
C4	4	2	0	2.153	0.972	0.536	–0.815	***	5	0	0	4.313	1.000	0.768	–0.302	ns
C5	3	2	0	1.058	0.056	0.055	–0.022	ns	4	1	1	1.871	0.529	0.465	–0.138	ns
C21 L1	2	1	1	1.009	0.009	0.009	–0.005	ns	2	0	0	1.192	0.176	0.161	–0.097	ns
C21 L2	10	7	3	2.936	0.944	0.659	–0.431	***	10	4	6	5.294	0.533	0.811	0.342	***
C33	4	2	1	2.069	0.935	0.517	–0.809	***	2	0	0	1.993	0.471	0.498	0.056	ns
Mean	3.619	1.952	0.714	1.710	0.559	0.326	–0.411		3.667	0.619	0.809	2.307	0.471	0.472	–0.004	
Total	76	41	15						77	21	17					

Na, Number of alleles; N ≤ 0.05, number of alleles with frequency less than five; Private, number of private alleles; Ne, effective number of alleles; Ho, observed heterozygosity; He, expected heterozygosity; Fis, fixation index; ns, non-significant *P < 0.05, ***P < 0.001.

**FIGURE 3:**
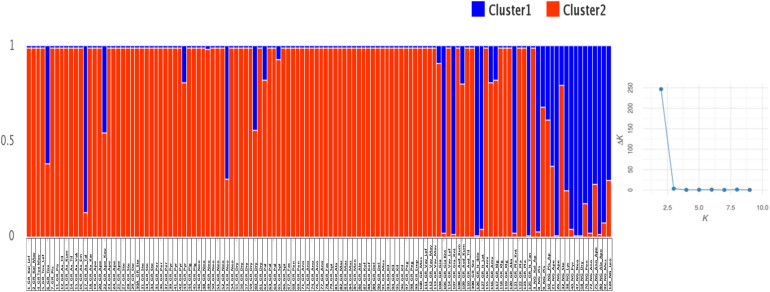
Structure analysis of the 124 carob trees with optimum cluster *k* = 2. The first component of the id code refers to tree number, the second to grafted (GR) or non-grafted trees (NG), the third to tree location and the fourth to the variety identification by farmers (Supplementary File 1 for code interpretation).

**FIGURE 4:**
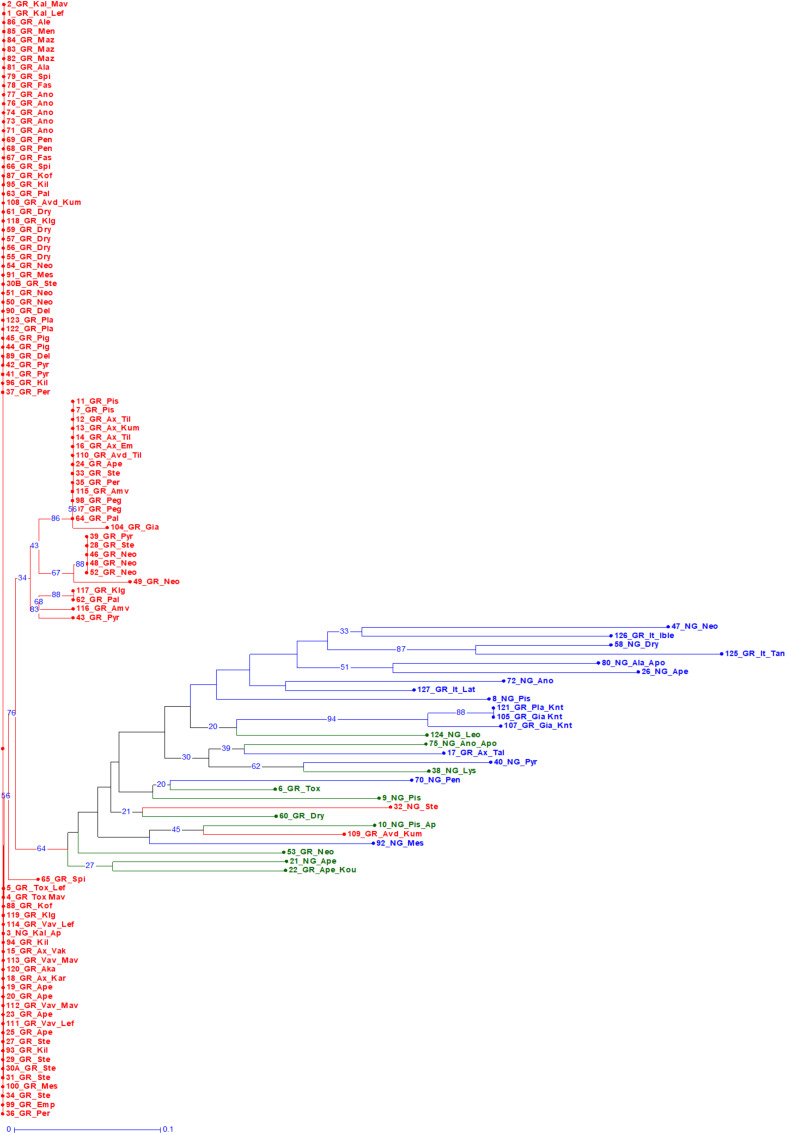
Bootstrapped Neighbor Joining dendrogram of the 124 trees. Trees grouped into clusters (G) and (N) of the structure analysis are shown in red and blue color, respectively. Admixture (A) genotypes are in green color. The first component of the id code refers to tree number, the second to grafted (GR) or non-grafted trees (NG), the third to tree location, and the fourth to the variety identification by farmers (Supplementary File 1 for code interpretation).

A Bayesian approach was employed to examine population structure. The analysis grouped trees into two clusters (Figure 3). Ninety-eight trees had membership coefficient over 0.8 to cluster 1 (hereafter cluster G), 16 trees had membership coefficient over 0.8 to cluster 2 (hereafter cluster N) and 10 trees were admixtures (hereafter A). The vast majority of grafted trees grouped into cluster (G), along with two non-grafted trees. Contrary, the majority of non-grafted trees grouped into cluster (N) along with the three Italian varieties, the three trees identified by farmers as “Kountourka” and one grafted tree from the DOA plantations with the id name “Tala.” Admixtures (A) were four grafted and six non-grafted trees (Figure 2). Clustering of trees according to the Neighbor-Joining dendrogram was consistent with the results of the structure analysis (Figure 4). Grafted trees were discriminated from non-grafted trees, with one exception, a tree identified by farmers as “Apostoliki—id code 3_NG_Kal_Ap.” The vast majority of the grafted trees, identified by farmers and stakeholders with the names “Imeri,” “Tillyrias,” “Koumpota,” “Empa,” “Lefkaritiki,” “Mavroteratsia,” “Vaklaes,” “Sarakina,” “Eftakoili,” “Koutsoulia,” and “Koumparkes” were genetically identical or similar. Most of these trees exhibited similar pod morphological traits, except from “Koutsoulia” and “Koumparkes.” The latter entries produced very short pods. Despite genetic similarity, it is noteworthy that differences on morphological and compositional traits were identified in orchards where “Lefkaritiki” and “Mavroteratsia” were grown side by side (Supplementary File 3). The grafted trees that were nonetheless genetically differentiated from the core of the grafted trees produced pods of variable and atypical morphology (e.g., very long “17_Gr_Ax_Talas”, intermediate “6_Gr_Tox” and very short “22_Gr_Ape_Koutsoulia.” Entries identified as “Kountourka” constitute a divergent genepool of grafted material. Genetic variation was also present within “Kountourka.” These entries produced pods with distinct morphological traits compared to the other grafted material (Supplementary File 4).

### Variation for Morphological and Compositional Traits

Descriptive statistics of the morphological and compositional traits are shown in Tables 3, 4 and their frequency plots are presented in Supplementary File 5. Average pod weight, length and width over seasons were 17.35 (g), 15.24 (cm), and 23.25 (mm), respectively. Variation was higher for the ratio seeds/pod weight and for pod weight (Table 3). Average powder color lightness (L^∗^) and total sugars content over seasons were 67.99 and 45.27 g 100 g^–1^ dw, respectively. Variation was very low for these traits. Sucrose and mallic acid were the predominant soluble carbohydrate and organic acid, respectively. Variation was lower for sucrose and fructose compared to glucose. Phosphorous (PO_4_) and potassium were the major metals detected in carob pods. Average DPPH, FRAP and total phenolics extracted with MeOH-HCL over seasons were 9.04 g AE kg^–1^ dw, 45.76 g AE kg^–1^ dw and 29.85 g GAE kg^–1^ dw, respectively. Gallic acid was the main phenolic compound detected followed by the flavonoid kaempferol-7-O-glucoside. Variation was higher for total phenolic content, condensed tannins, antioxidant activity and individual polyphenols compared to the other compositional and morphological traits (Table 4).

**TABLE 3:** Means, standard errors (Std Error), Coefficients of Variation (CV), maximum and minimum values for morphological traits, percentage of sum of squares in ANOVA explained by the agro-environmental zones (Sign Env) and structure clusters (Sign Str).

Trait	N	Mean	Std Error	CV	Minimum	Maximum	Sign Env	Sign Str
Pod weight (g) (2018)	98	16.83	0.468	27.51	5.806	29.11	11.1	12.3**
Pod weight (g) (2019)	99	17.88	0.553	30.75	4.777	31.81	22.5**	14.4**
Ratio seeds/pod weight (2018)	98	8.758	0.283	31.94	3.975	16.72	10.6	55.4***
Ratio seeds/pod weight (2019)	100	8.532	0.288	33.75	4.508	17.08	9.4	48.0***
Length (cm) (2018)	98	15.12	0.237	15.50	7.540	20.41	12.7	0.6
Length (cm) (2019)	100	15.36	0.235	15.32	7.133	20.00	17.8*	0.3
Width (mm) (2018)	98	23.33	0.187	7.95	16.57	26.13	8.1	28.8***
Width (mm) (2019)	100	23.18	0.211	9.11	15.82	28.10	12.5	30.9***
Edge thickness (mm) (2018)	98	9.829	0.142	14.34	5.342	12.88	13.1	25.9***
Edge thickness (mm) (2019)	100	10.10	0.128	12.65	6.534	12.82	14.8	43.6***
Groove thickness (mm) (2018)	98	8.798	0.133	15.00	4.488	11.76	12.1	14.5**
Groove thickness (mm) (2019)	100	9.259	0.116	12.57	5.202	11.71	7.5	43.9***
Ratio edge/groove (2018)	98	1.138	0.011	9.77	0.826	1.443	22.8**	9.4*
Ratio edge/groove (2019)	100	1.103	0.008	7.23	0.899	1.330	30.7***	4.6
Valley thickness (mm) (2019)	98	5.735	0.071	12.23	2.577	8.097	9.1	6.6*
Valley thickness (mm) (2019)	100	8.849	0.093	10.51	6.969	12.03	7.6	4.0
Average seed weight (g) (2018)	98	0.202	0.004	20.93	0.126	0.492	11.1	9.8**
Average seed weight (g) (2019)	100	0.172	0.002	11.72	0.120	0.220	10.9	1.2
L* pod external (2018)	98	23.75	0.156	6.51	19.99	28.97	21.9**	7.7*
L* seed (2018)	98	34.21	0.311	9.00	27.25	39.78	23.5**	0.5

*p < 0.05, **p < 0.01, ***p < 0.0001.

**TABLE 4:** Means, standard errors (Std Error), Coefficients of Variation (CV), maximum and minimum values for compositional traits, percentage of sum of squares in ANOVA explained by the agro-environmental zones (Sign Env) and structure clusters (Sign Str).

Trait	N	Mean	Std. Error	CV	Minimum	Maximum	Sign Env	Sign Str
L* powder (2018)	98	67.26	0.583	8.59	49.10	76.78	67.0***	7.3*
L* powder (2019)	100	68.72	0.501	7.29	52.47	77.33	39.9***	16.2**
Sucrose (g 100 g^–1^ dw) (2018)	98	34.19	0.497	14.39	12.10	43.40	21.8**	20.1***
Sucrose (g 100 g^–1^ dw) (2019)	100	30.12	0.444	14.75	12.60	38.00	13.5	16.0**
Fructose (g 100 g^–1^ dw (2018)	98	9.983	0.132	13.13	6.600	14.00	29.9***	7.8*
Fructose (g 100 g^–1^ dw) (2019)	100	7.449	0.125	16.75	4.600	12.00	17.4*	3.5
Glucose (g 100 g^–1^ dw) (2018)	98	5.245	0.131	24.71	2.400	10.20	48.3***	9.5**
Glucose (g 100 g^–1^ dw) (2019)	100	3.547	0.109	30.71	1.500	9.300	22.6**	3.1
Total Sugars (g 100 g^–1^ dw) (2018)	98	49.42	0.430	8.62	27.80	57.00	9.8	29.7***
Total Sugars (g 100 g^–1^ dw) (2019)	100	41.12	0.401	9.75	30.50	49.20	10.2	20.4***
Malic acid (g kg^–1^ dw) (2018)	98	5.873	0.160	26.98	1.495	10.71	37.2***	1.6
Citric acid (g kg^–1^ dw) (2018)	98	1.598	0.047	29.15	0.351	2.897	36.5***	10.4**
Oxalic acid (g kg^–1^ dw) (2018)	98	0.304	0.005	15.89	0.206	0.442	29.0***	1.7
Total organic acid (g kg^–1^ dw) (2018)	98	7.774	0.180	22.89	2.568	13.45	41.1***	0.1
Protein% (w/w) (2018)	98	4.691	0.095	20.07	2.179	7.868	18.6*	1.2
PO_4_ (g kg^–1^ dw) (2018)	98	1.213	0.036	29.65	0.568	2.611	16.1*	2.3
SO_4_ (g kg^–1^ dw) (2018)	98	0.505	0.035	68.76	0.083	1.648	9.6	4.3
NH_4_ (g kg^–1^ dw) (2018)	97	0.147	0.013	86.76	0.000	0.749	13.4	4.2
NO_3_ (g kg^–1^ dw) (2018)	98	0.066	0.003	47.62	0.020	0.197	16.8	0.4
K (g kg^–1^ dw) (2018)	98	12.14	0.267	21.74	4.546	19.89	10	0.04
Cl (g kg^–1^ dw) (2018)	98	1.982	0.056	27.91	1.080	3.994	30.2***	5.0
Ca (g kg^–1^ dw) (2018)	98	1.640	0.066	39.82	0.231	4.453	28.9***	2.3
Mg (g kg^–1^ dw) (2018)	98	1.013	0.029	28.76	0.218	1.913	18.0*	1.3
Na (g kg^–1^ dw) (2018)	98	0.216	0.014	62.96	0.047	0.843	18.9*	5.2
DPPH (g AE kg^–1^ dw) (2018)	98	9.899	0.437	43.65	2.100	24.10	44.5***	11.6**
DPPH (g AE kg^–1^ dw) (2019)	100	8.181	0.629	76.85	0.250	39.04	32.6***	9.9**
FRAP (g AE kg^–1^ dw) (2018)	98	53.61	2.703	49.91	11.00	130.5	66.8***	3.6
FRAP (g AE kg^–1^ dw) (2019)	100	37.91	1.886	49.76	11.49	86.98	53.4***	0.5
Total Phenolics (MeOH-HCL) (g GAE kg^–1^ dw) (2018)	98	34.07	1.864	54.15	7.500	85.40	71.2***	2.8
Total Phenolics (MeOH-HCL) (g GAE kg^–1^ dw) (2019)	100	25.63	1.805	70.42	3.021	99.18	50.4***	1.7
Total Phenolics—(MeOH-acetate) (g GAE kg^–1^ dw) (2018)	98	11.08	0.429	38.26	3.700	29.10	39.8***	11.6**
Condensed tannins (mg Catechin g^–1^ dw) (2018)	98	47.89	2.871	59.38	5.000	108.0	68.9***	0.8
Condensed tannins (mg Catechin g^–1^ dw) (2019)	100	24.98	2.361	94.35	0.000	97.80	56.1***	0.03
Gallic acid (μg g^–1^ dw) (2018)	98	959.6	56.64	58.43	52.89	4015	24.3**	5.9
Kaempferol-7-O-glucoside (μg g^–1^ dw) (2018)	98	74.87	6.598	87.24	0.000	438.6	4.8	13.2**
Gallocatechin (μg g^–1^ dw) (2018)	98	11.90	0.240	19.94	0.000	19.93	8.4	0.8
Naringenin diglucoside (μg g^–1^ dw) (2018)	98	11.76	0.758	63.80	0.000	31.34	33.9***	0.6
Gallocatechin gallate (μg g^–1^ dw) (2018)	98	10.23	0.943	91.34	0.000	41.30	24.3**	2.5
Catechin (μg g^–1^ dw) (2018)	98	9.479	0.894	93.38	0.000	66.08	15.1	0.1
Isovitexin (μg g^–1^ dw) (2018)	98	5.580	0.261	46.36	0.426	13.31	7.1	1.7
Luteolin-7-O-glucoside (μg g^–1^ dw) (2018)	98	5.041	0.374	73.77	0.000	17.13	13.8	0.1
Epigallocatechin gallate (μg g^–1^ dw) (2018)	98	3.213	0.089	27.51	0.000	6.228	5.8	0.7
Methyl gallate (μg g^–1^ dw) (2018)	98	2.882	0.214	73.43	0.000	10.99	31.4***	7.2*
Epicatechin (μg g^–1^ dw) (2018)	98	1.508	0.036	23.81	0.000	2.867	13.8	0.6
Vitexin (μg g^–1^ dw) (2018)	98	0.955	0.076	79.19	0.436	5.045	8.6	0.3
Total (μg g^–1^ dw) (2018)	98	1097	59.48	53.67	89.91	4139	22.1**	7.4*

*p < 0.05, **p < 0.01, ***p < 0.0001.

### Genotypic and Environmental Effect on Morphological and Compositional Traits

**FIGURE 5:**
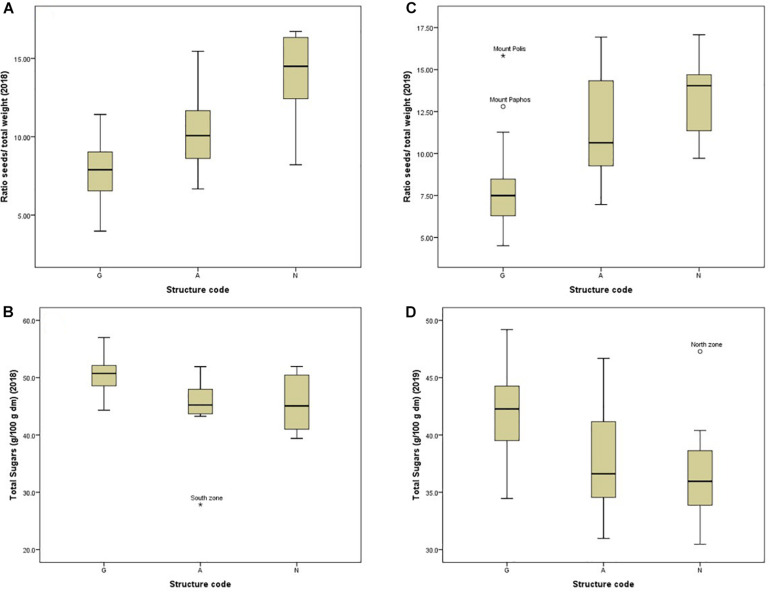
Box plots showing the variation between and within structure clusters for the ratio seeds- to-pod weight **(A,B)** and for total sugars **(C,D)** during the first and the second season.

**FIGURE 6:**
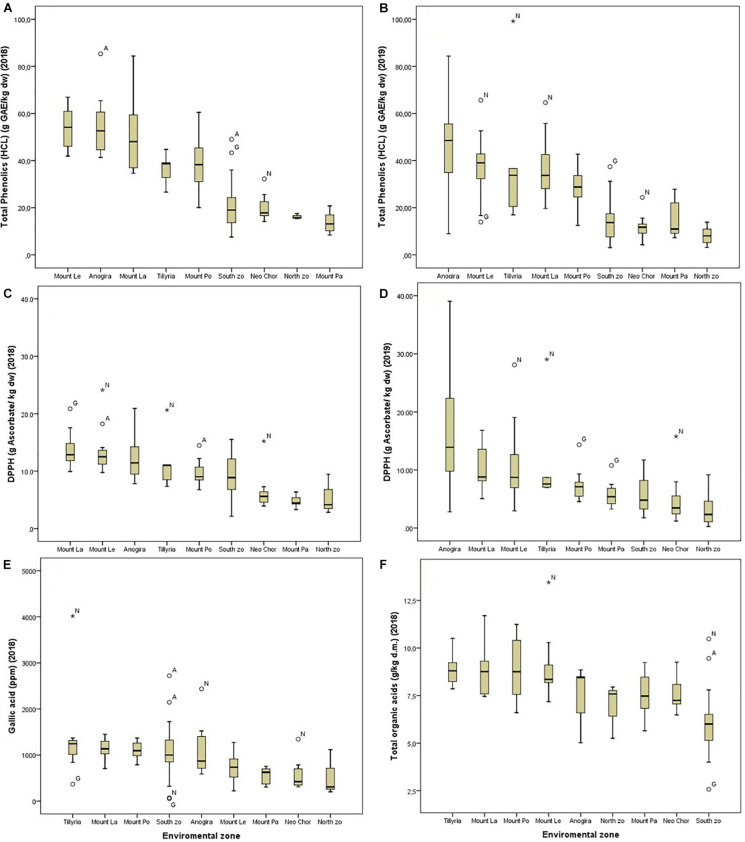
Box plots showing the variation across different agro-environmental zones for total phenolics extracted with MeOH-HCL in 2018 **(A)** and in 2019 **(B)** for antioxidant activity (DPPH) in 2018 **(C)** and in 2019 **(D)** and for gallic acid concentration **(E)** and total organic acids **(F)** in 2018. Outlier codes refer to their structure cluster.

ANOVA showed that genotype had a stronger effect on morphological traits, particularly on the ratio seeds/pod weight, pod width and thickness (Table 3). Descriptive statistics for each structure cluster and agro-environmental zone are presented in Supplementary File 6. Pods of trees grouped into cluster (G) had lower seeds/pod weight ratio (Figure 5), they were wider, thicker and heavier. On the other hand, the effect of the agro-environment was stronger on most of the compositional traits, particularly on total phenolics extracted with MeOH-HCL, FRAP, condensed tannins and powder color and to lesser extent on DPPH, total phenolics extracted with MeOH-acetate, malic, citric, oxalic, total organic acids, gallic acid, PO_4_, Cl, Ca, Mg, and Na (Table 4). Variation in total phenolics extracted with MeOH-HCL, antioxidant activity, gallic acid and total organic acids concentrations between the different agro-environmental zones is shown in Figure 6. Most of the outliers are trees grouped in cluster N or they were admixtures (A). Concerning carbohydrates, the agro-environmental effect was stronger on reducing sugars fructose and glucose, the genotypic effect was stronger on total sugars, while genotypic and agro-environmental effects on sucrose were equal (Table 4). Carobs from trees grouped into cluster G had higher total sugars than those grouped into cluster N or they were admixtures (Figure 5B).

### Seasonal Effect on Morphological and Compositional Traits

Overall, the seasonal effect was statistically significant for all traits except for the ratio seeds/pod weight, pod length and width (Table 5). Pods were heavier and thicker in 2019, while the concentrations of soluble carbohydrates, condensed tannins, total phenolics and antioxidant activity (DPPH and FRAP) were lower (Tables 3, 4). Pearson correlations between seasons were strong and positive for most morphological traits and slightly lower for powder color, DPPH, FRAP, condensed tannins, total phenolics extracted with MeOH-HCL and total sugars. Among carbohydrates, sucrose had the strongest correlation, while fructose and glucose correlations were lower and similar. The correlations for valley thickness and average seed weight were non-significant.

**TABLE 5:** Pearson correlation coefficients (r), significance of correlations (Sig) and significance of t test for traits between seasons.

Trait	*r*	Sig.	*t*-test
Pod weight	0.809	***	**
Ratio seeds/pod weight	0.781	***	ns
Length	0.743	***	ns
Width	0.754	***	ns
Edge thickness	0.764	***	**
Groove thickness	0.646	***	***
Ratio edge/Groove	0.580	***	***
Valley thickness	–0.184	ns	***
Average seed weight	0.015	ns	***
L* powder	0.693	***	*
Sucrose	0.586	***	***
Fructose	0.389	***	***
Glucose	0.383	***	***
Total sugars	0.517	***	***
DPPH	0.685	***	**
FRAP	0.699	***	***
Total Phenolics (MeOH-HCL)	0.663	***	***
Condensed tannins	0.612	***	***

*p < 0.05, **p < 0.01, ***p < 0.0001.

### Hierarchical Cluster Analysis on Phenotypic Traits

**FIGURE 7:**
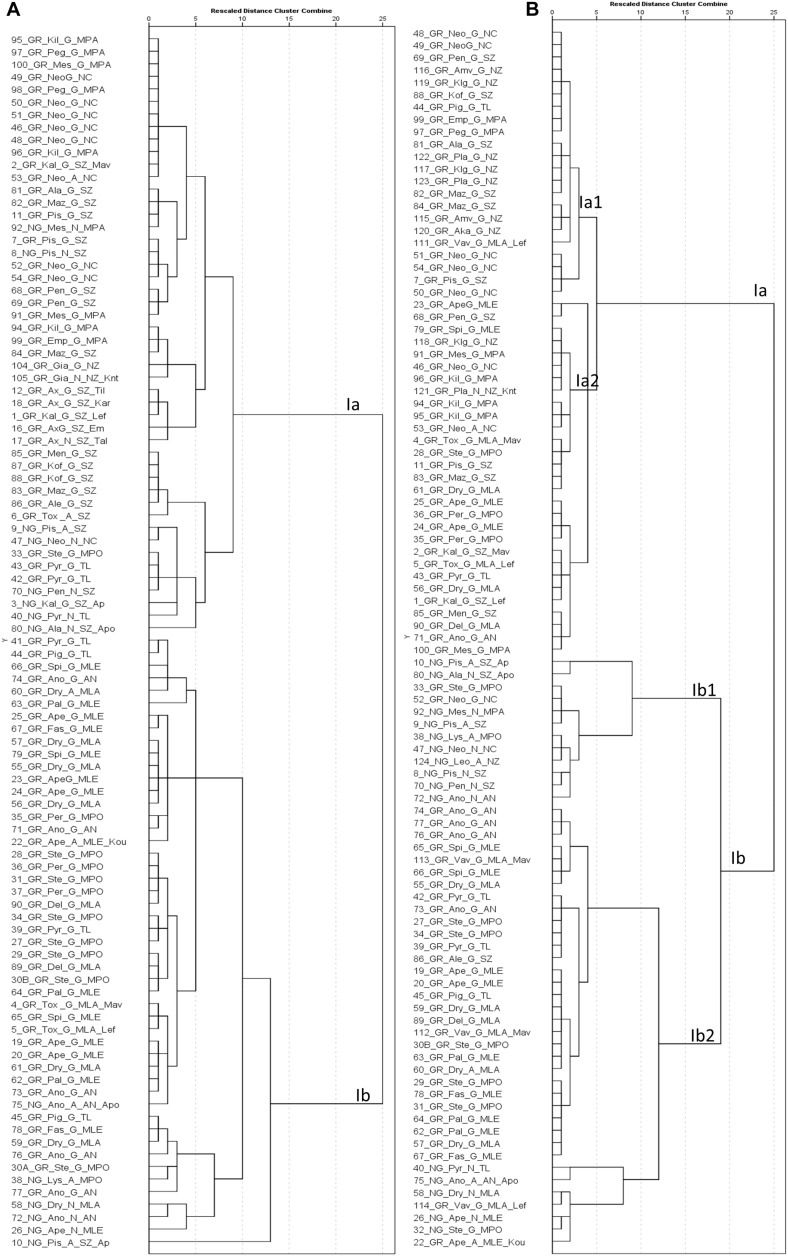
Hierarchical cluster analysis based on phenotypic traits measured in 2018 **(A)** and 2019 **(B)**. The first component of the id code refers to tree number, the second to grafted (GR) or non-grafted trees (NG), the third to tree location, the fourth to the structure cluster, the fifth to the agro-environmental zone where the tree grows and the sixth to the variety identification by farmers (Supplementary File 1 for code interpretation).

In 2018, hierarchical cluster analysis grouped trees in two major clusters of phenotypes (Figure 7A). The first cluster (Ia) contained the trees collected from the agro-environmental zones Neo Chorio, mountainous Paphos, north and south zones. The second cluster (Ib) contained exclusively trees collected from Anogira, mountainous Polis, Larnaca, Lemesos and Tillyria with one exception (10_NG_Pis_A_SZ_Ap). In 2019, cluster Ia1 contained trees collected almost exclusively from the agro-environmental zones Neo Chorio, mountainous Paphos, north and south zone while cluster Ib2 contained trees collected from mountainous Polis, Larnaca, Lemesos, and Tillyria (Figure 7B). Cluster Ib1 contained trees either collected from the latter agro-environmental zones or trees from the former agro-environmental zones that were grouped into cluster N or were admixtures.

## Discussion

### SSRs Revealed Low Genetic Diversity Within Grafted Trees

Genetic diversity was assessed in a set of 107 grafted and 17 non-grafted trees distributed in nine agro-environmental zones of Cyprus, using 18 microsatellites. The SSRs discriminated 36 genotypes out of the 124 trees examined. They distinguished all non-grafted trees from grafted trees, of which one common genotype appeared in high frequency. La Malfa et al. (2014) reported that SSRs failed to distinguish some accessions presenting limited phenotypic variation. Likewise, SSRs in the current study did not discriminate neighboring trees showing slightly different morphological and compositional traits, as in the case of “Mavroteratsia” and “Lefkaritiki.” Moreover, SSRs in some cases failed to distinguish accessions of grafted trees bearing pods of distinctive characters, e.g., “Koutsoulia” or “Koumbarkes” having very short pods (< 10 cm). Although morphological and compositional differentiation can also be attributed to other factors, such as rootstock-scion interaction (Emmanouilidou and Kyriacou, 2017), the discriminating ability of published SSRs for phenotypically similar grafted trees remains a concern. It can be concluded that, SSRs can be effectively used for the assessment of carob genetic diversity relating to non-grafted genetic material (Viruel et al., 2018); however, other molecular techniques (e.g., next-generation genotyping) should also be employed to examine in more detail the genetic diversity of grafted material (La Malfa et al., 2014; Viruel et al., 2018; Di Guardo et al., 2019).

Grafted carob is a vegetatively (asexually) propagated tree with expectedly lower genetic diversity than an annual crop in similar environments, e.g., Cypriot durum wheat landraces (Kyratzis et al., 2019). Moreover, carob genetic diversity is also lower than that of olive (Anestiadou et al., 2017; Emmanouilidou et al., 2018) and pomegranate Cypriot genetic resources (Kyriacou et al., 2020). The lower genetic diversity compared to other tree species from Cyprus is consistent with the scenario of declining carob genetic resources purported by Viruel et al. (2018). Genetic variability within the cultivated genepool can be increased through hybridization between local or/and foreign material, and subsequent selection by farmers (Tous et al., 2013; Anestiadou et al., 2017). Farmers’ selection criteria for carob were rather limited (Batlle and Tous, 1997; Tous et al., 2013), therefore selection pressure for limited morphological and compositional traits could result in further declination of genetic diversity within the cultivated carob genepool compared to other tree species (e.g., pomegranate Kyriacou et al., 2020) presenting wider phenotypic variability, ease of propagation (e.g., by cuttings) and complexity of fruit quality traits. Furthermore, the carob population suffered a severe decline over the last 50 years mainly owing to the cultivation of irrigated cash crops, illicit logging and land development, as evidenced in the substantial reduction of the carob cultivated area (Davies, 1970). For example, Tillyria was historically the most famous area for carob production in Cyprus (Gennadius, 1902), hence the variety “Tillyria” is synonymous with this area (Orphanos and Papaconstantinou, 1969); nowadays however, only few remaining scattered carob trees testify the area’s past agricultural landscape.

The genetic diversity detected in the present study and the percentage of discriminated genotypes were lower than previous works on carob (La Malfa et al., 2014; Viruel et al., 2018; Di Guardo et al., 2019), which can be attributed to the fact that sampling was limited to Cyprus and emphasis was placed on grafted material. Italian varieties were genetically divergent from the core of the Cypriot grafted germplasm; however, they shared, to very large extent, polymorphism that exists within the non-grafted Cypriot material supporting the concept of multiple domestications of the carob tree from native populations throughout the Mediterranean basin (Viruel et al., 2018).

Non-grafted trees grouped at a different structure cluster than the core of the grafted trees or they were admixtures. Despite the smaller sample size, genetic diversity among non-grafted trees was higher, Fis was close to zero and most loci did not show significant deviation from Hardy Weinberg equilibrium indicating that contrary to grafted material wild populations were under random mating. Nevertheless, gene flow exists between grafted and non-grafted genetic material. Grafted trees producing pods of variable morphology were grouped as admixtures in structure analysis confirming that admixture is an important component of carob genetic diversity (Di Guardo et al., 2019). As carob is a cross pollinated species and grafted trees are not easily distinguished from non-grafted ones, farmers unconsciously select trees from segregating material once they discern desirable pod traits. Lower prices obtained in some geographical areas due to inferior quality (Gennadius, 1902) could also drive farmers to experiment with selection of local phenotypes, as was observed in the Karpasia peninsula (Ticho, 1959). This “new” genetic diversity appears with low frequency, less than 1% indicating a low selection pressure from farmers. Therefore, extensive sampling is needed to track genetic diversity within grafted carob genetic resources.

### Genetic Analysis Suggests the Existence of Two Carob Landraces

Based on the definition of landrace by Villa et al. (2005), it can be concluded that two carob landraces exist in Cyprus grouped into separate genetic clusters, each composed of genetically similar or identical trees. The first landrace, which predominates on the island, encompasses the genetic material known under the local names “Mavroteratsia,” “Lefkaritiki,” “Tillyria,” “Koumpota,” “Imeri,” “Kamateri,” “Saradjina,” and “Vakles.” The second landrace, which is locally common to Karpasia peninsula, encompasses the genetic material known under the local name “Kountourka.” These two landraces produced pods of distinct morphology and they can be easily distinguished also by the different tree morphology (Orphanos and Papaconstantinou, 1969). A slightly different compositional profile for these two landraces was also reported (Davies et al., 1971). Our results demonstrated that morphological and compositional differentiation of these two landraces are aligned with genetic differentiation. Contrary, Di Guardo et al. (2019) found that “Tillyria” and “Kountourka” are genetically similar varieties. This discrepancy, which is likely due to mislabeling of Cypriot accessions, confirms that passport data of *ex situ* collections should be treated with caution (Kyratzis et al., 2019). Mislabeling was also identified within the DoA plantations which provide budding wood for propagation purposes. Therefore, genetic identification of mother plants providing budding wood to nurseries is crucial to ensure access to certified material of the desirable genotype.

### Morphological Traits Are Predominantly Under Genetic Control While Compositional Traits Are Mainly Under Agro-Environmental Control

Morphological traits, carbohydrate and protein content were comparable to those reported by Orphanos and Papaconstantinou (1969). In support of Tetik et al. (2011), variation in morphological traits was found larger for the ratio seed/pod weight and pod mass. Sucrose was the major sugar present while fructose and glucose were detected in lower concentrations, in agreement with Biner et al. (2007). The variation in total sugars was similar to the variation in soluble solids found in Turkish carobs (Tetik et al., 2011). Our results further confirm that carob is a rich source of polyphenols, including high concentrations of condensed tannins (Avallone et al., 1997; El Bouzdoudi et al., 2016). Gallic acid was the major phenolic compound and mallic acid the major organic acid (Ayaz et al., 2007; Farag et al., 2019). Contrary to Portuguese carobs (Custodio et al., 2011), catechin appeared at very low concentration. Potassium was the major metal detected in the pulp accompanied by considerable concentration of calcium, phosphorus and magnesium (Oziyci et al., 2014; Goulas et al., 2016).

Morphological and compositional traits of carobs can be affected by genotype (Barracosa et al., 2007), gender (Custodio et al., 2011), climatic conditions (Tous et al., 2013), harvest stage (Benchikh et al., 2014; Farag et al., 2019), soil conditions, and season (Correia et al., 2018). In the present study, genotypic effect was high for pod morphological traits, particularly for the seeds/pod weight ratio, pod width and thickness, as well as total sugars. These traits were under strong selection pressure from farmers (Batlle and Tous, 1997; Tetik et al., 2011). During ripening, there is a slight reduction of glucose and fructose and a progressive accumulation of sucrose, with a significant effect of the environment on their relative concentrations (Othmen et al., 2019). Our results suggest that sucrose accumulation can be equally affected by the genotype and the agro-environment, contrary to the accumulation of monosaccharides that is mainly under agro-environmental control. Accordingly, Biner et al. (2007) found higher sucrose concentration on grafted material compared to wild trees, while differences in fructose and glucose were non-significant. Farag et al. (2019) reported that the discrimination of carob samples of different geographical origins is more effective when based on monosaccharaides, which can be attributed to the high agro-environmental effect on their accumulation. It may be inferred that the potential for sugar accumulation is to an extent genetically regulated, however the final concentration of total sugars deposited in the pod and especially the relative concentrations of reducing sugars to sucrose are influenced by the agro-environmental effect on the ripening process.

Agro-environment had a strong effect on total phenolic content, antioxidant activity, condensed tannins and color of the carob powder, and to lesser extent on polyphenolic composition. Farag et al. (2019) reported that tannins and flavonoids were the major compounds for discriminating carob samples of different origin. Previous studies reported significant differences between varieties or environments for condensed tannins, total phenolic content and antioxidant activity (Avallone et al., 1997; Custodio et al., 2011; Benchikh et al., 2014; Nahla, 2014; El Bouzdoudi et al., 2016; Othmen et al., 2019), nevertheless the reported variation between varieties or environments was larger in the present study, likely due to the extensive sampling performed in different agro-environments. Despite the high agro-environmental effect, substantial variation was also observed within each agro-environment (Figure 6) indicating the presence of genetic variation for these traits. However, as selection of promising genotypes is time consuming, describing the environments enhancing the accumulation of these secondary metabolites would be a faster approach to improve bioactive content than promoting the cultivation of improved genotypes rich on these compounds.

### Morphological and Compositional Traits Vary Significantly Between Seasons

Carob pod size can be affected by environmental factors as well as level of pollination and fruit set (Batlle and Tous, 1997). In the present study, the effect of both the agro-environment and season on pod size were non-significant and strong correlations were observed between seasons indicating that pod morphology is rather stable, mainly driven by genetic factors. Average rainfall during the first season was slightly lower than normal (90%) while the second season was exceptionally rainy (158% of normal; Cyprus Meteorological, 2020 access 27/05/2020). As favorable environmental conditions increase the heritability of agronomic traits (Blum, 2010), it is likely that the environmental and seasonal effects on pod morphology diminished. Carob pods were heavier and thicker in 2019, contrary to the non-significant differences in pod length and width implying that carob trees modulate carob pod weight by adjusting pod thickness.

Seasonal effect was significant on antioxidant activity, total phenolic content and condensed tannins, which were lower during the 2nd season. The exceptionally high precipitation of the 2nd season likely reduced water stress resulting in lower phenolic content and antioxidant activity TE (Tavarini et al., 2011; Nasrabadi et al., 2019). The strong correlations between seasons for antioxidant activity, total phenolic content and condensed tannins suggest the presence of low season-by-tree interactions. Contrary, the lower correlations between seasons for carbohydrates indicates the presence of a strong season-by-tree interaction for these compounds, particularly for glucose and fructose and to lesser extent on sucrose. The lower concentration of carbohydrates in the season where precipitation was exceptionally high and average carob production was lower (Personal communication with stakeholders 2019) might be attributed to physiological factors related to alternate bearing (Von Haselberg, 1996), which triggered carob trees to invest carbohydrates in vegetative growth and development rather than storage in pods. The season-by-tree interactions for carbohydrates can be explained in the context that alternate bearing is not ubiquitous in the carob population (Rosenstock et al., 2010), thus in the same year both “on-season” and “off-season” trees are encountered.

### Two Major Agro-Environmental Zones Shape the Compositional Profile of Carobs in Cyprus

Carob producing areas in Cyprus can be divided into two major agro-environmental zones based on the effect of the agro-environment on compositional traits and the overall performance depicted by hierarchical cluster analysis. The first major zone encompasses the agro-environmental zones mountainous Anogira, Larnaca, Polis, Lemesos and Tillyria where carobs are characterized by lighter-colored pulp (higher powder L^∗^), higher antioxidant activity, higher concentration of total phenolics, condensed tannins, organic acids, calcium and sucrose. These compounds are highly significant for the characterization of carob as a functional food (Rasheed et al., 2019). The second major zone encompasses the agro-environmental zones south and north, mountainous Paphos and Neo Chorio where carobs are characterized by darker carob pulp, lower antioxidant activity, lower concentration of total phenolics, condensed tannins, organic acids and sucrose, and higher concentration of fructose and glucose. The vast majority of grafted trees in the two major zones were genetically similar or identical and were grouped in the same genetic cluster. Since the standardization of the crude pod material is important for the processing industry, the narrow genetic basis of grafted material in Cyprus implies that the local processing industry should categorize this material based on whether it was collected from grafted or non-grafted trees and according to the agro-environmental zone of origin.

Trees grouped into structure cluster (N) or those that were admixtures (A) behaved in many cases as outliers within particular environmental zones (Figure 6); this was further evident in their grouping in the hierarchical cluster analysis (Figure 7). Therefore, compositional traits are modulated by the agro-environment and the genotype. The present study also emphasizes that seasonal effect on compositional traits should also be considered. Despite previous efforts to categorize carobs according to their geographical origin (Farag et al., 2019; Kokkinofta et al., 2020), these works suffer from the limited description either of the genetic profile or the environmental conditions where trees were grown, moreover they lack replication in time. These limitations that are not easily manageable for an underutilized tree crop like carob, warrant international cooperation for the establishment of *ex situ* collections of the same divergent genetic material in different agro-environmental zones to allow accurate assessment of the genotypic, agro-environmental and seasonal effects on carob compositional traits and to facilitate association mapping studies.

## Conclusion

Genotyping analysis of carob genetic resources, using microsatellites revealed low genetic diversity levels within Cypriot grafted germplasm. Two carob landraces were identified: “Tillyria” and “Kountourka” consisted of genetically similar or identical trees. “Tillyria” predominates on the island while “Kountourka” is locally common to the Karpasia peninsula. Genetically divergent genotypes from the abovementioned landraces were identified, which appeared though with very low frequency, indicating a low selection pressure from farmers. Morphological traits, particularly the seeds/pod weight ratio, pod width and thickness, and the total sugar content, were mainly under genetic control. On the other hand, agro-environmental conditions had a strong effect on compositional traits, particularly on total phenolics, antioxidant activity, condensed tannins and color of the carob powder. Concerning sugar profile, the agro-environmental effect was stronger on fructose and glucose, while sucrose was equally affected by genotype and agro-environment. Significant differences between seasons for morphological and compositional traits were observed revealing the existence of considerable seasonal effect. Correlations between seasons were stronger for morphological traits, intermediate for powder color, antioxidant activity, total phenolic content and condensed tannins, and lower for carbohydrates suggesting the existence of a strong tree-by-season interaction on sugar content. Based on all the above findings, Cyprus can be divided to two major agro-environmental zones that modulate to a great extent the compositional properties of the carob pulp.

## Data Availability Statement

The raw data supporting the conclusions of this article will be made available by the authors, without undue reservation.

## Ethics Statement

The studies involving human participants were reviewed and approved by the ethics committee of the Agricultural Research Institute of the Cyprus Ministry of Agriculture, Rural Development and Environment. The participants provided written informed consent to participate in this study.

## Author Contributions

AK, MK, and CA: conceptualization, methodology, and data curation. AK, MK, CA, LP, GG, and YR: formal analysis, writing—original draft preparation, and writing—review and editing. MK, LP, GG, and YR: resources. AK and MK: supervision and project administration. All authors have read and agreed to the published version of the manuscript.

## Conflict of Interest

The authors declare that the research was conducted in the absence of any commercial or financial relationships that could be construed as a potential conflict of interest.
