# Supplementary material for: Pod Morphology, Primary and Secondary Metabolite Profiles in Non-grafted and Grafted Carob Germplasm Are Configured by Agro-Environmental Zone, Genotype, and Growing Season
Source: Front Plant Sci. 2021 Jan 13;11:612376. doi: 10.3389/fpls.2020.612376 (PMC7838365; doi:10.3389/fpls.2020.612376)
Supplement: Supplementary file 2 [file Table_2.DOCX]

**Supplementary file 2:** List of primers used in the present study, sequence, type of fluoroform and annealing temperature –Ta (^o^C) for the reactions.

| **Primer** | **Sequence 5'-3'** | **Repeat Motif** | **Fluoroform** | **Ta (^o^C)** | **Reference** |
| --- | --- | --- | --- | --- | --- |
| Cesi 21 cttt7_FW | GGGGAAAACAACCAATATAGTTA | CTTT7 | FAM | 57 | Malfa et al., 2014 |
| Cesi 21 cttt7_RV | AGGAGATCGAGCGTATGCAG |  |  |  |  |
| Cesi 187 at15_FW | ATACTGGGCGTTCTTTGCTT | AT15 | FAM | 57 |  |
| Cesi 187 at15_RV | ATTATCTCTTGCTTTGTGGTCCT |  |  |  |  |
| Cesi 1187 at9_FW | TTCTCGTCGCCCAAACTG | AT9 | FAM | 55 |  |
| Cesi 1187 at9_RV | CTCCCTCATCTCCTTCGTTG |  |  |  |  |
| Cesi 98 gct6_FW | GCCACCACTTTGAAGGAAGA | GCT6 | FAM | 57 |  |
| Cesi 98 gct6_RV | GCTAGAAGCAGGAGCAGGAG |  |  |  |  |
| Cesi 15 aaatag4_FW | GACGGTGGAAGGCAACCT | AAATAG4 | FAM | 57 |  |
| Cesi 15 aaatag4_RV | GCTCGCTTGGGGAGTGTA |  |  |  |  |
| Cesi 976 ta5tg6_FW | TCCTGAAGGCTGAAGATGATG | TA5TG6 | FAM | 57 |  |
| Cesi 976 ta5tg66_RV | CAAACCAATGAAGGGCTCTA |  |  |  |  |
| Cesi 74 ta7_FW | AACGCAAACCTCAGCATCAT | TA7 | FAM | 57 |  |
| Cesi 74 ta7_RV | AAGGCAAGTGGGAGACACAC |  |  |  |  |
| Cesi 17 tta7_FW | AAATGCAACAAAGATGACACG | TTA7 | FAM | 56 |  |
| Cesi 17 tta7_RV | GAAGAAAGCTCGGCCTCTG |  |  |  |  |
| C8_FW | TTCTACCGCACTTCAGCCTC | ATCT & T | FAM | 56 | Viruel et al., 2018 |
| C8_RV | TCTTGCTTCACTACCTCCCC |  |  |  |  |
| C10_FW | TGGATCACAACTTAAAAGCCCG | TTCT | FAM | 56 |  |
| C10_RV | TGCCGAAATCGCTCGTAAAC |  |  |  |  |
| C22_FW | GCTCCATTCTCTGTCAATCACC | TCTT | FAM | 56 |  |
| C22_RV | GACCAGCGGTGAGAAATTACC |  |  |  |  |
| C23_FW | AATTTGGCGATAGCAGAAGC | AGAA | FAM | 56 |  |
| C23_RV | GCATGTCCAGATTCCATGATTTC |  |  |  |  |
| C29_FW | TGGATGTCCTTTAAACTGAACGAG | AGAA | FAM | 56 |  |
| C29_RV | CGGTGAGGGGTATCAGGATG |  |  |  |  |
| C31_FW | TCAAGGGTTGCATTCGGAG | AAAG | FAM | 56 |  |
| C31_RV | GCTTTCCCAAGTGCTTCAAC |  |  |  |  |
| C-04_FW | GTCTGTTGAGTAGCCACCAC | TAGA | FAM | 56 |  |
| C-04_RV | AGCATCTATCTCTTCCCTCGC |  |  |  |  |
| C-05_FW | TACATCTGCGCTGCCATTTC | AGAT | FAM | 56 |  |
| C-05_RV | AGTTTCTAGCTTGGTCTTTCGC |  |  |  |  |
| C-21_FW | CGTGAATCCTTTGACGGGC | TTC & T | HEX | 56 |  |
| C-21_RV | ACACAGTCTATTTTTGTACCCAATC |  |  |  |  |
| C-33_FW | TGCATGTGTTTCATGTACGC | ATGT | HEX | 56 |  |
| C-33_RV | TGACATCTCCACTAAGCCTCC |  |  |  |  |
