# Supplementary material for: Pod Morphology, Primary and Secondary Metabolite Profiles in Non-grafted and Grafted Carob Germplasm Are Configured by Agro-Environmental Zone, Genotype, and Growing Season
Source: Front Plant Sci. 2021 Jan 13;11:612376. doi: 10.3389/fpls.2020.612376 (PMC7838365; doi:10.3389/fpls.2020.612376)
Supplement: Supplementary file 3 [file Table_3.DOCX]

**Supplementary file 3:** Morphological and compositional differences between ‘Mavroteratsia’ and ‘Lefkaritiki’. Observations are from carob orchards where neighboring tress of ‘Mavroteratsia’ and ‘Lefkaritiki’ were identified by farmers. Therefore, mean value depicts the differences between them irrespective of the agro-environment, and standard error (Std error) shows the variation due to the different agro-environments of the orchards. Two orchards were evaluated in 2018 (n=2) and four orchards in 2019 (n=4).  **
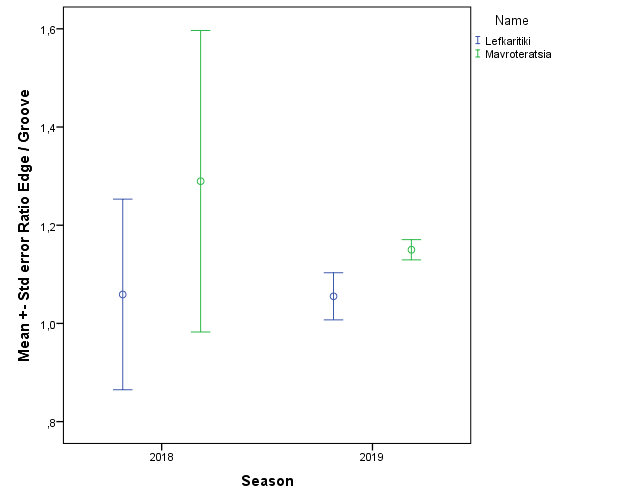
**

**
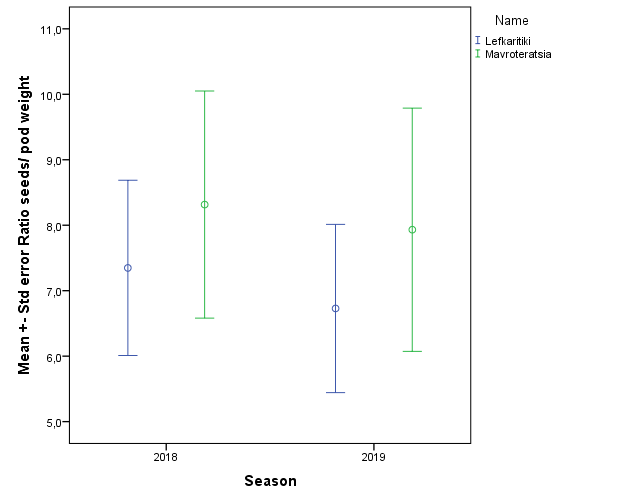
**


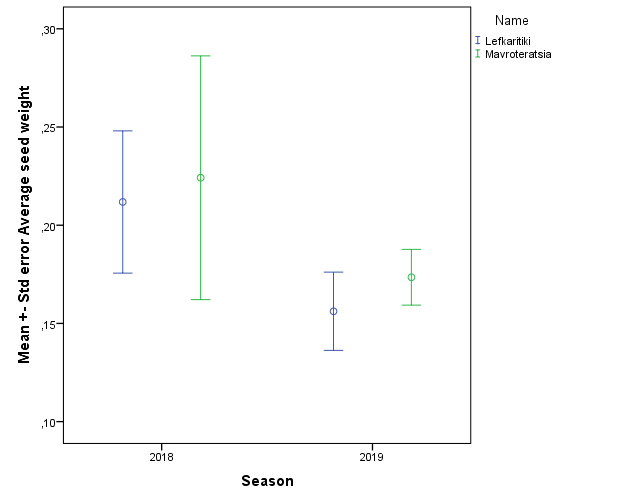


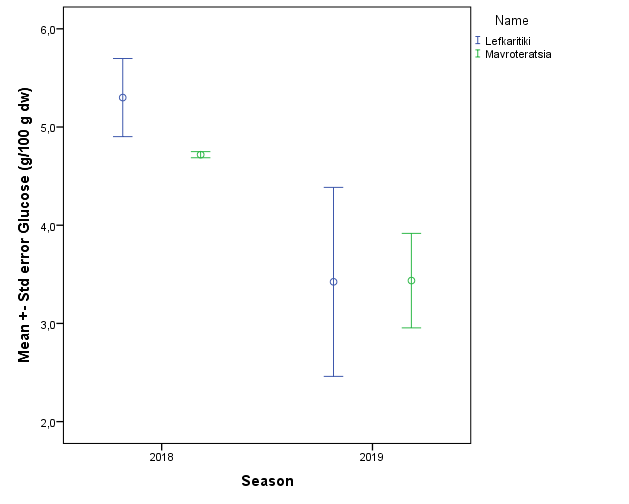

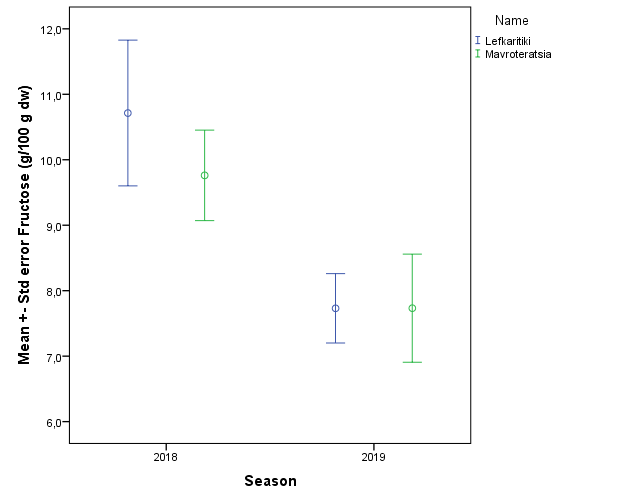

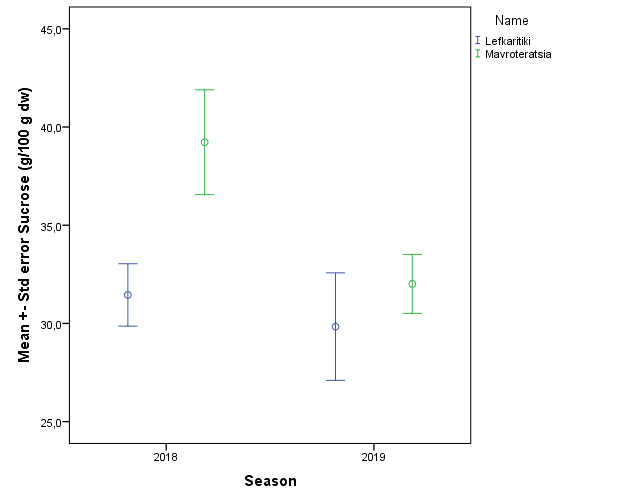


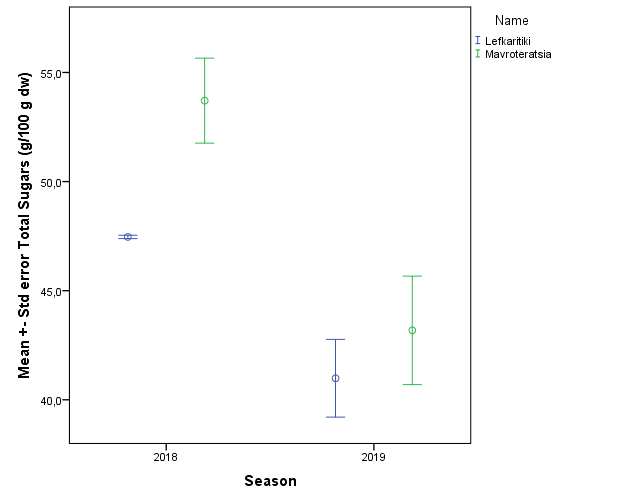


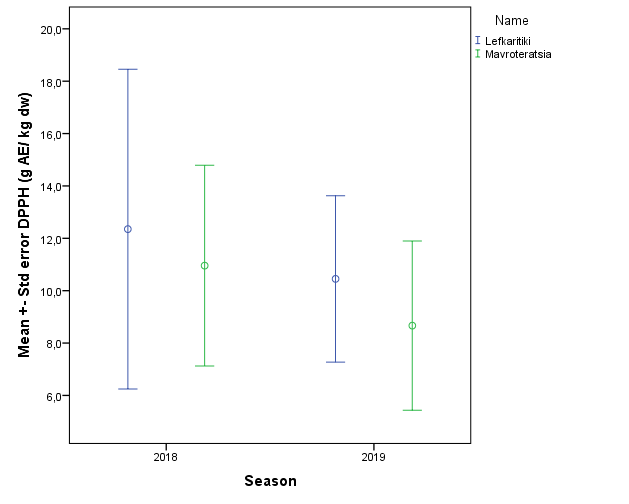


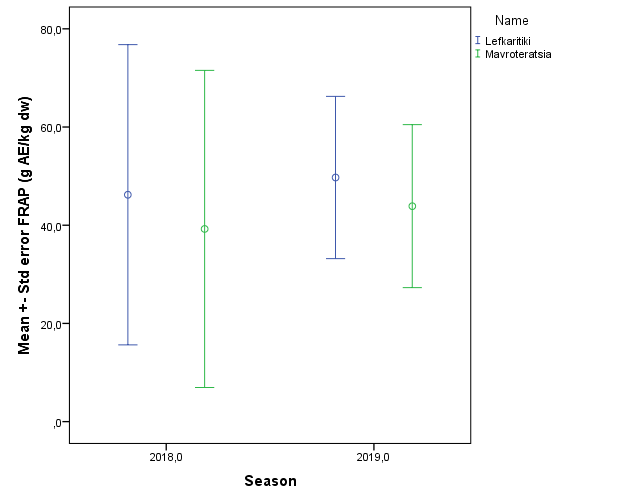


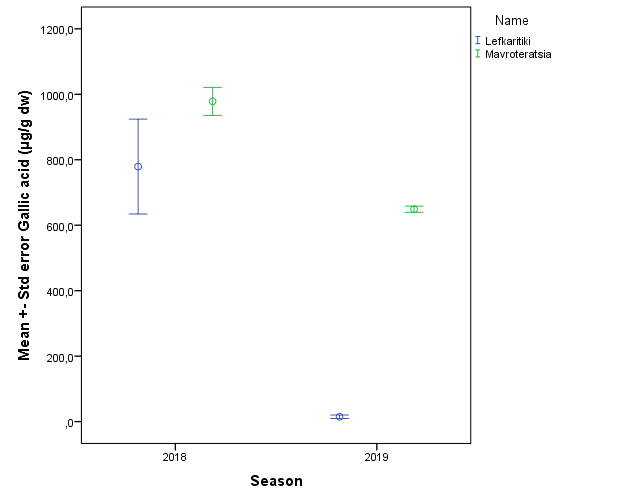


Carobs of ‘Mavroteratsia’ and ‘Lefkaritiki’ from neighboring trees from Kalavasos area. The photos were taken on the 9^th^ of July 2019.
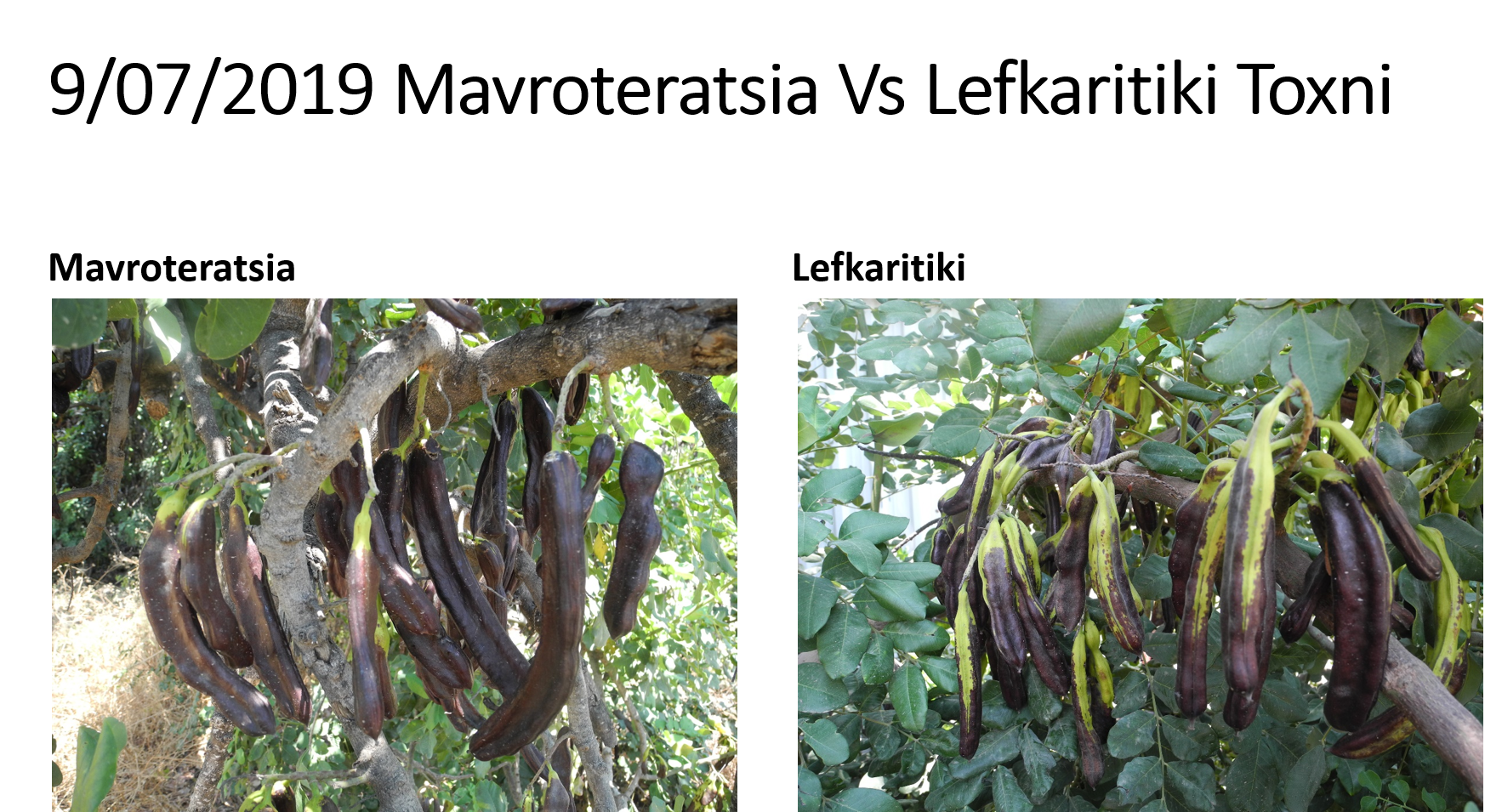


Pods of ‘Lefkaritiki’ (right) and ‘Mavroteratsia’ (left) from neighboring trees from Kalavasos area collected in 2019. Pods of ‘Mavroteratsia’ were slightly curved compared to ‘Lefkaritiki’. The color of the pods was black to ‘Mavroteratsia’ and brown to ‘Lefkaritiki’. Edge was more intense to ‘Lefkaritiki’. ‘Mavroteratsia’ pods usually beard bulges towards edge side. Pod weight, length and width were equal. Edge side thickness was higher than groove side in ‘Mavroteratsia’.

| 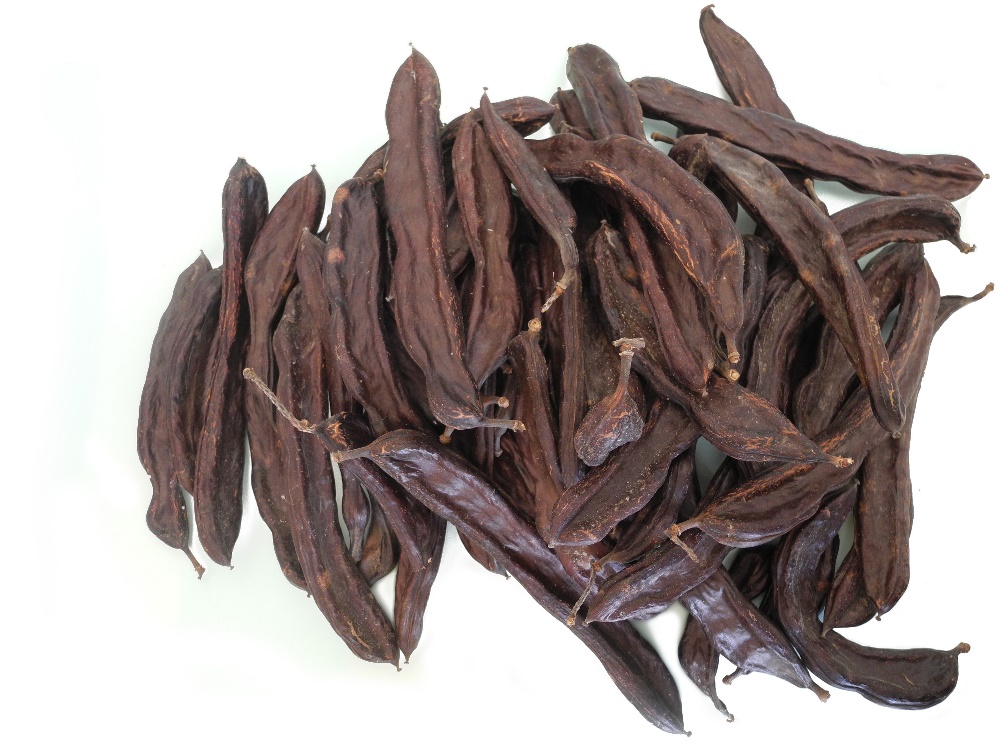 | 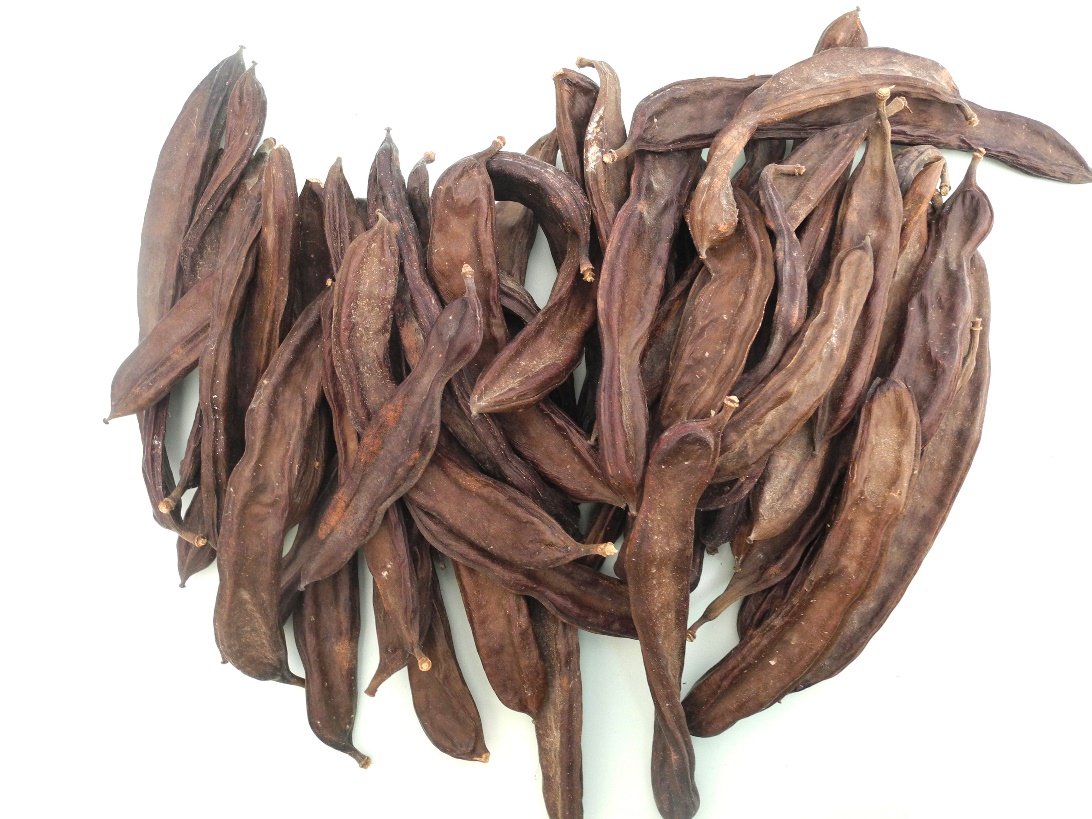 |
| --- | --- |
| 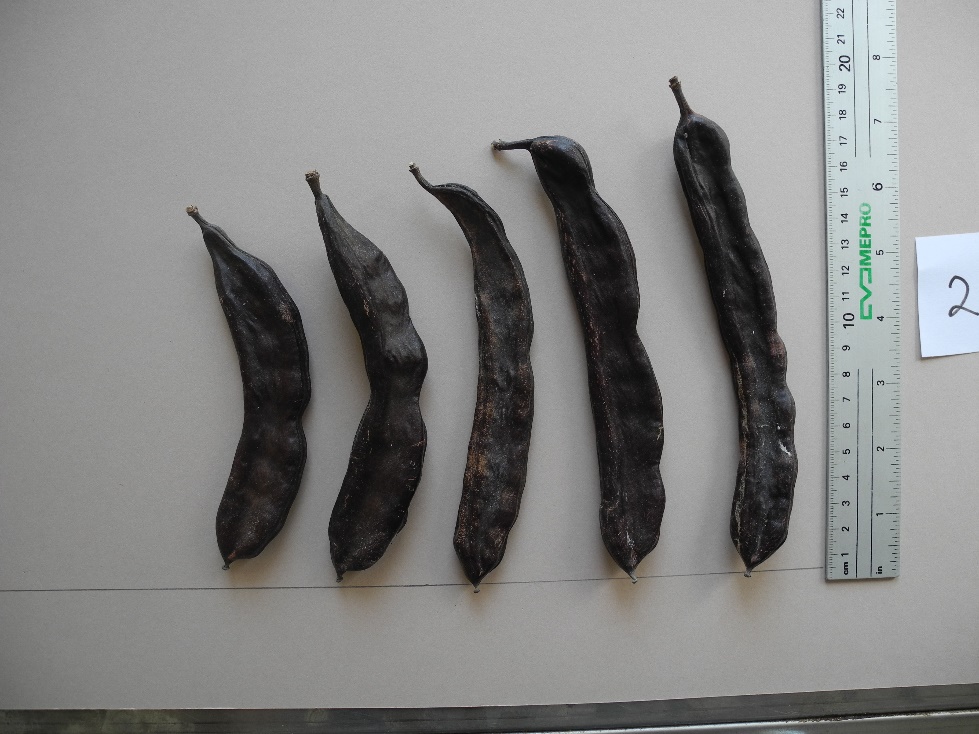 | 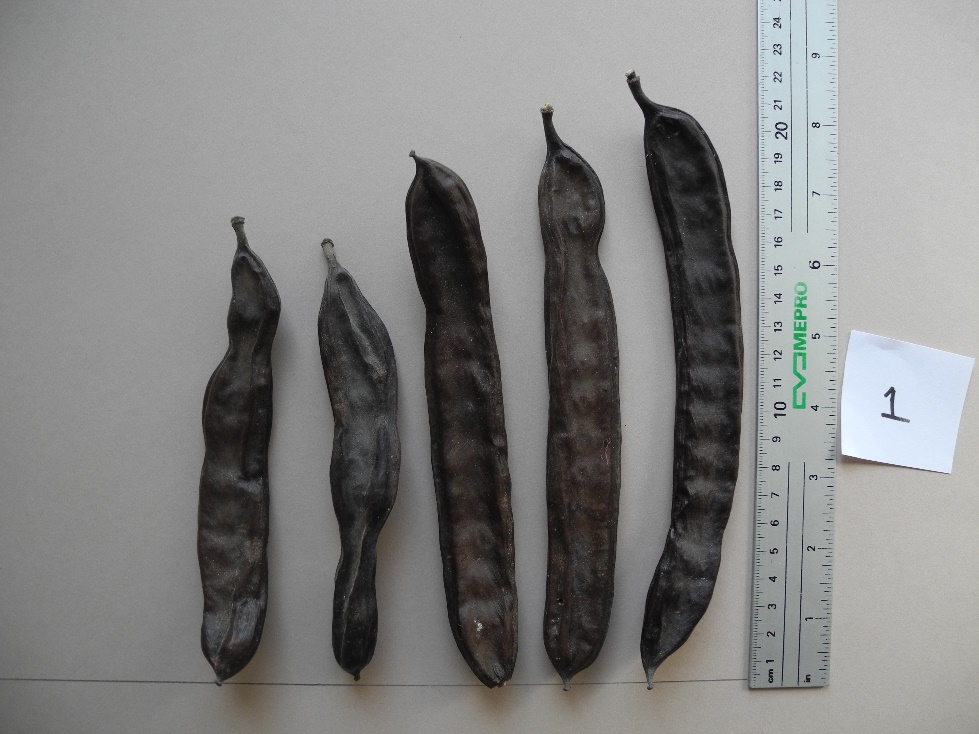 |
