# Supplementary material for: Pod Morphology, Primary and Secondary Metabolite Profiles in Non-grafted and Grafted Carob Germplasm Are Configured by Agro-Environmental Zone, Genotype, and Growing Season
Source: Front Plant Sci. 2021 Jan 13;11:612376. doi: 10.3389/fpls.2020.612376 (PMC7838365; doi:10.3389/fpls.2020.612376)

**Supplementary file 4:** Differences on pod morphology between landraces ‘Tillyria’ and ‘Kountourka’. Means and standard errors are shown from pod measurements of nine trees identified as ‘Tillyria’ and three trees identified as ‘Kountourka’. All trees were grown at the north zone.


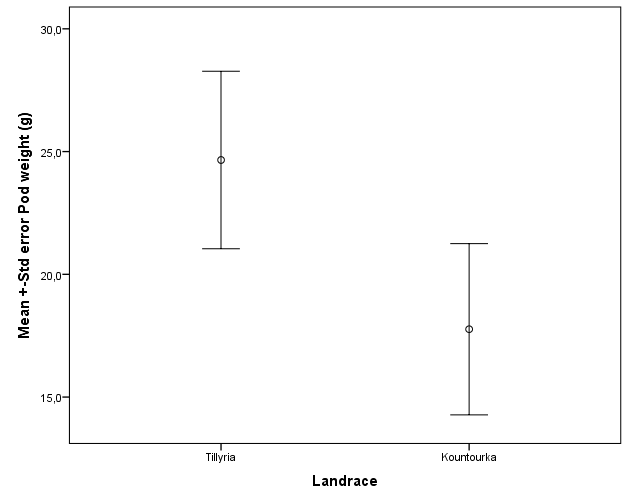

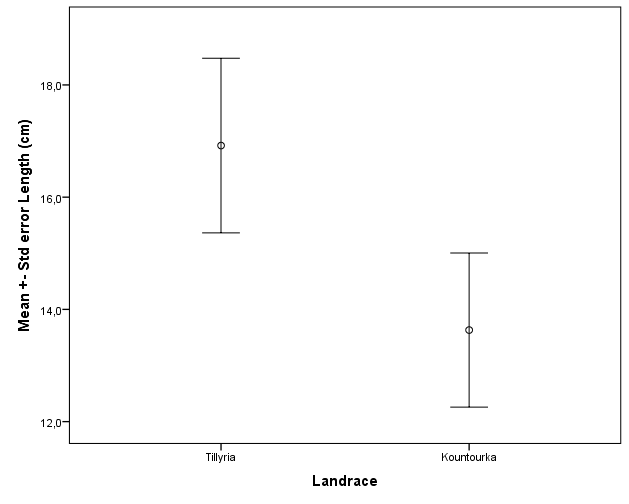

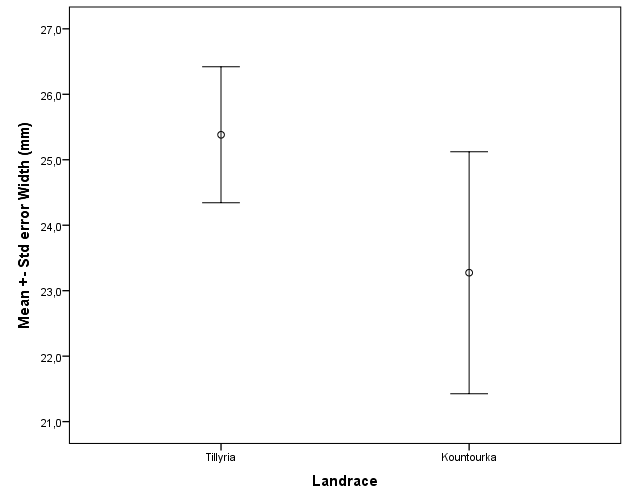

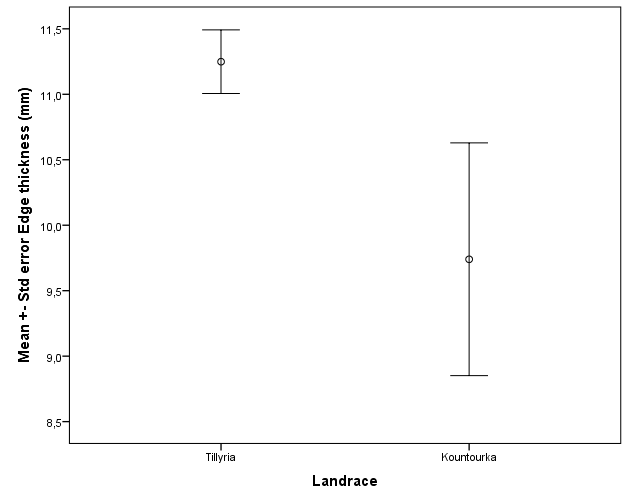


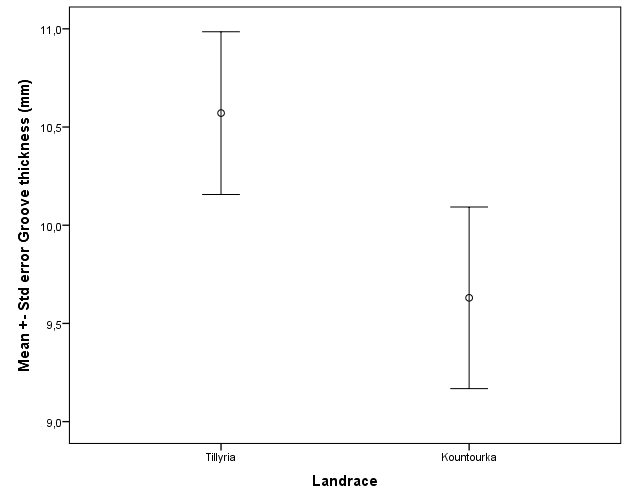


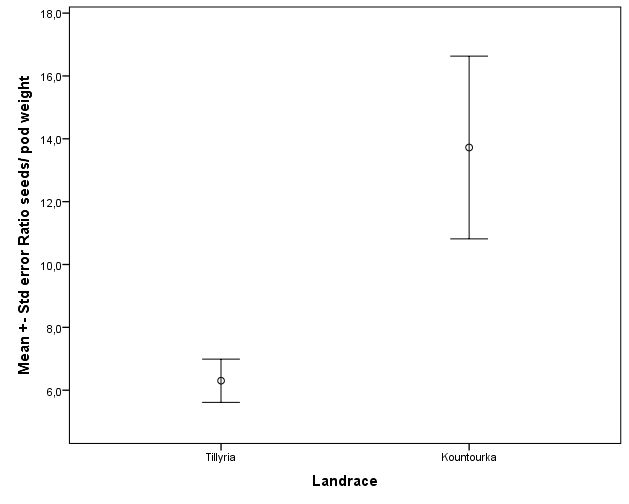

Supplement: Supplementary file 4 [file Table_4.DOCX]
